# Supplementary material for: Effects of organic-inorganic complex fertilizer on the growth and physiological characteristics of ‘Qi-Nan’ agarwood from Aquilaria sinensis (Lour.)
Source: PLoS One. 2025 Apr 4;20(4):e0320766. doi: 10.1371/journal.pone.0320766 (PMC11970636; doi:10.1371/journal.pone.0320766)
Supplement: S1 File — (DOCX) [file pone.0320766.s001.docx]

Effects of organic-inorganic complex fertilizer on the growth and physiological characteristics of 'Qi-Nan' agarwood from Aquilaria sinensis (Lour.)

**Jingyue Huang^1,3^, Yunlin Fu^1^, Zhu Yu^2^, Xueting Li^1^, Siyu Zheng^1^, Fenyong Tang^2^ and Penglian Wei^1^ ***

1 Guangxi Colleges and Universities Key Laboratory for Cultivation and Utilization of Subtropical Forest Plantation, College of Forestry, Guangxi University, Nanning, China, 2 Guangxi Forest Inventory & Planning Institute, Nanning, China, 3 Guangxi Gaofeng State Owned Forest Farm.

* weipenglian@gxu.edu.cn

| **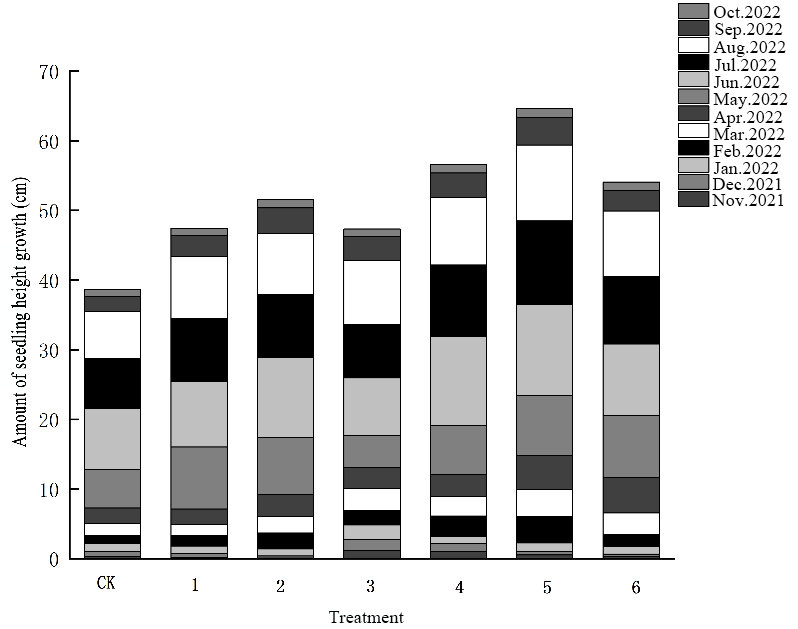** |
| --- |

Fig 1. Variation in seedling height growth of ‘Qi-Nan’ agarwood seedlings

Tab 1. Dynamics of seedling height growth of ‘Qi-Nan’ agarwood seedlings under different fertilization rates (Units: cm)

| **Month** | **Treatment** | | | | | | | **ANOVA** |
| --- | --- | --- | --- | --- | --- | --- | --- | --- |
|  | **CK** | **1** | **2** | **3** | **4** | **5** | **6** |  |
| Nov. | 17.9 | 17.2 | 17.1 | 17.5 | 17.8 | 18 | 17.7 | >0.05 |
| Dec. | 18.2 | 17.6 | 17.7 | 18.5 | 18.9 | 18.1 | 17.9 | <0.05 |
| Jan. | 18.9 | 17.9 | 18.1 | 19.6 | 20.5 | 18.4 | 18.4 | <0.05 |
| Feb. | 20 | 18.9 | 19.4 | 20.7 | 22.6 | 19.4 | 19.5 | <0.001 |
| Mar. | 21.3 | 20.7 | 23.1 | 23.6 | 24.6 | 21.7 | 21.1 | <0.001 |
| Apr. | 22.9 | 23.8 | 27.1 | 26.4 | 27.9 | 24 | 22.6 | <0.001 |
| May. | 25.2 | 28.9 | 32 | 29.6 | 30.9 | 27.3 | 24.9 | <0.001 |
| Jun. | 30.7 | 37.8 | 40.6 | 36.6 | 35.4 | 35.4 | 33.8 | <0.001 |
| Jul. | 39.5 | 46.3 | 53.7 | 49.4 | 43.7 | 46.9 | 43.2 | <0.001 |
| Aug. | 46.7 | 56 | 65.7 | 59.7 | 51.4 | 56 | 52.2 | <0.001 |
| Sep. | 53.5 | 65.4 | 76.5 | 69.3 | 60.6 | 64.7 | 61.1 | <0.001 |
| Oct. | 55.6 | 68.4 | 80.6 | 72.9 | 64 | 68.4 | 64.1 | <0.001 |
| Nov. | 56.6 | 69.6 | 81.8 | 74.1 | 65.1 | 69.5 | 65.2 | <0.001 |
| Total increment | 38.7±3.2De | 52.4±4.9Cbc | 64.7±5.6Aa | 56.6±3.2Bb | 47.3±4.2Ccd | 51.5±5.0Cbd | 47.5±3.8Ccd | <0.001 |
| Relative increment | 100.00% | 135.40% | 167.30% | 146.40% | 122.50% | 133.30% | 122.70% | <0.001 |
| P | 0.00 | 0.00 | 0.00 | 0.00 | 0.00 | 0.00 | 0.00 |  |

Tab. 2 Multiple-factor comparison of treatments main effects on plant height growth of month

| **Treatment** | **CK** | **1** | **2** | **3** | **4** | **5** | **6** |
| --- | --- | --- | --- | --- | --- | --- | --- |
| PLH | 3.22Dd | 4.36BCbc | 5.39Aa | 4.72Bb | 3.94Cc | 4.29BCbc | 3.96Cc |

Tab. 3 Multiple comparison of monthly main effect on plant height growth of ‘Qi-Nan ’

| **Treatment** | **Feb.** | **May.** | **Aug.** | **Nov.** |
| --- | --- | --- | --- | --- |
| PLH | 0.84Dd | 2.82Cc | 8.90Aa | 4.51Bb |

Raw data for Fig 1, Tab 1,Tab. 2 and Tab. 3

| 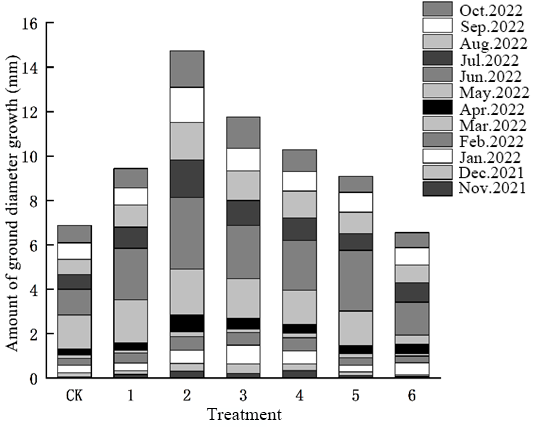 |
| --- |

Fig 2. Variation in ground diameter growth of ‘Qi-Nan’ agarwood seedling

Tab 4. Dynamics of ground diameter growth of ‘Qi-Nan’ agarwood seedlings under different fertilization rates (Units: mm)

| **Month** | **Treatment** | | | | | | | **ANOVA** |
| --- | --- | --- | --- | --- | --- | --- | --- | --- |
|  | **CK** | **1** | **2** | **3** | **4** | **5** | **6** |  |
| Nov. | 5.36 | 4.76 | 4.89 | 5.26 | 4.53 | 5.01 | 4.86 | <0.05 |
| Dec. | 5.42 | 4.92 | 5.2 | 5.47 | 4.87 | 5.13 | 4.93 | >0.05 |
| Jan. | 5.6 | 5.09 | 5.55 | 5.9 | 5.17 | 5.29 | 5.01 | <0.05 |
| Feb. | 5.94 | 5.44 | 6.15 | 6.74 | 5.75 | 5.59 | 5.55 | <0.001 |
| Mar. | 6.25 | 5.89 | 6.76 | 7.31 | 6.35 | 5.93 | 5.85 | <0.001 |
| Apr. | 6.4 | 6.02 | 6.99 | 7.48 | 6.55 | 6.11 | 5.97 | <0.001 |
| May. | 6.66 | 6.35 | 7.74 | 7.94 | 6.95 | 6.47 | 6.38 | <0.001 |
| Jun. | 8.2 | 8.28 | 9.79 | 9.74 | 8.48 | 8.03 | 6.8 | <0.001 |
| Jul. | 9.36 | 10.6 | 13.03 | 12.13 | 10.73 | 10.76 | 8.28 | <0.001 |
| Aug. | 10.02 | 11.56 | 14.71 | 13.26 | 11.74 | 11.52 | 9.16 | <0.001 |
| Sep. | 10.71 | 12.55 | 16.4 | 14.59 | 12.95 | 12.48 | 9.95 | <0.001 |
| Oct. | 11.45 | 13.32 | 17.98 | 15.61 | 13.82 | 13.37 | 10.73 | <0.001 |
| Nov. | 12.24 | 14.2 | 19.63 | 17.02 | 14.81 | 14.1 | 11.41 | <0.001 |
| Total increment | 6.88±0.89d | 9.44±1.01c | 14.74±1.50a | 11.76±1.24b | 10.28±1.10bc | 9.09±0.76c | 6.55±0.75d | <0.001 |
| Relative increment | 100.00% | 137.21% | 214.24% | 170.93% | 149.42% | 132.12% | 95.20% | <0.001 |
| P | 0.00 | 0.00 | 0.00 | 0.00 | 0.00 | 0.00 | 0.00 |  |

Tab.5 Multiple-factor comparison of treatments main effects on diameter growth of month

| **Treatment** | **CK** | **1** | **2** | **3** | **4** | **5** | **6** |
| --- | --- | --- | --- | --- | --- | --- | --- |
| Grd | 0.58Ccd | 0.78BCbc | 1.22Aa | 0.98ABb | 0.8BC6b | 0.76BCbcd | 0.55Cd |

Tab.6 Multiple comparison of monthly main effect on diameter growth of ‘Qi-Nan ’

| **Treatment** | **Feb.** | **May.** | **Aug.** | **Nov.** |
| --- | --- | --- | --- | --- |
| Grd | 0.31Cc | 0.36Cc | 1.59Aa | 1.02Bb |

Raw data for Fig 2,Tab 4, Tab.5 and Tab.6

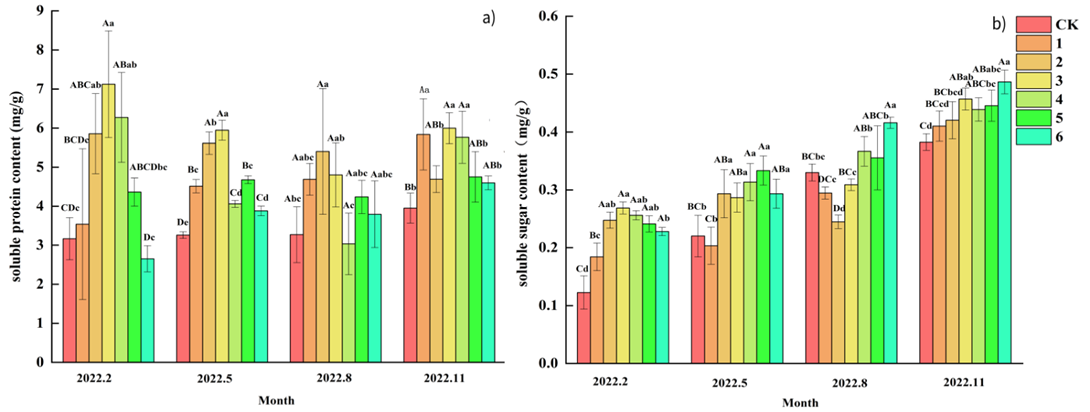


Fig 3. Effects of different fertilizer treatments on soluble protein (SP) and soluble sugar (SS) content of ‘Qi-Nan’ agarwood seedlings.

Raw data for Fig 3 SP

| **Treatment** | **Feb.** | **May.** | **Aug.** | **Nov.** |
| --- | --- | --- | --- | --- |
| ck | 3.76 | 3.34 | 3.04 | 4.31 |
| ck | 3.02 | 3.17 | 2.70 | 3.99 |
| ck | 2.71 | 3.26 | 4.07 | 3.55 |
| 1 | 2.66 | 4.69 | 5.06 | 5.07 |
| 1 | 2.20 | 4.35 | 4.75 | 6.85 |
| 1 | 5.75 | 4.50 | 4.26 | 5.59 |
| 2 | 6.70 | 5.49 | 4.36 | 4.76 |
| 2 | 6.15 | 5.94 | 4.59 | 4.32 |
| 2 | 4.72 | 5.41 | 7.26 | 4.99 |
| 3 | 8.63 | 5.76 | 4.01 | 5.66 |
| 3 | 6.75 | 6.24 | 4.74 | 6.43 |
| 3 | 5.99 | 5.84 | 5.65 | 5.90 |
| 4 | 6.74 | 4.06 | 2.23 | 5.05 |
| 4 | 4.96 | 4.15 | 3.06 | 5.86 |
| 4 | 7.11 | 3.97 | 3.81 | 6.38 |
| 5 | 4.77 | 4.67 | 3.74 | 5.36 |
| 5 | 4.12 | 4.78 | 4.47 | 4.82 |
| 5 | 4.19 | 4.58 | 4.49 | 4.07 |
| 6 | 2.98 | 3.98 | 4.10 | 4.41 |
| 6 | 2.66 | 3.74 | 4.45 | 4.61 |
| 6 | 2.31 | 3.92 | 2.83 | 4.77 |

Raw data for Fig 3 SS

| **Treatment** | **Feb.** | **May.** | **Aug.** | **Nov.** |
| --- | --- | --- | --- | --- |
| ck | 0.10 | 0.26 | 0.33 | 0.39 |
| ck | 0.15 | 0.19 | 0.34 | 0.37 |
| ck | 0.12 | 0.21 | 0.31 | 0.39 |
| 1 | 0.21 | 0.18 | 0.28 | 0.40 |
| 1 | 0.17 | 0.24 | 0.30 | 0.39 |
| 1 | 0.17 | 0.19 | 0.30 | 0.44 |
| 2 | 0.25 | 0.34 | 0.25 | 0.39 |
| 2 | 0.26 | 0.26 | 0.23 | 0.41 |
| 2 | 0.23 | 0.28 | 0.25 | 0.46 |
| 3 | 0.28 | 0.29 | 0.30 | 0.46 |
| 3 | 0.26 | 0.26 | 0.31 | 0.47 |
| 3 | 0.27 | 0.31 | 0.32 | 0.44 |
| 4 | 0.25 | 0.35 | 0.36 | 0.46 |
| 4 | 0.26 | 0.29 | 0.34 | 0.42 |
| 4 | 0.25 | 0.30 | 0.39 | 0.43 |
| 5 | 0.23 | 0.36 | 0.29 | 0.45 |
| 5 | 0.24 | 0.31 | 0.40 | 0.42 |
| 5 | 0.25 | 0.33 | 0.37 | 0.47 |
| 6 | 0.23 | 0.27 | 0.42 | 0.46 |
| 6 | 0.22 | 0.32 | 0.40 | 0.50 |
| 6 | 0.23 | 0.29 | 0.42 | 0.49 |

**
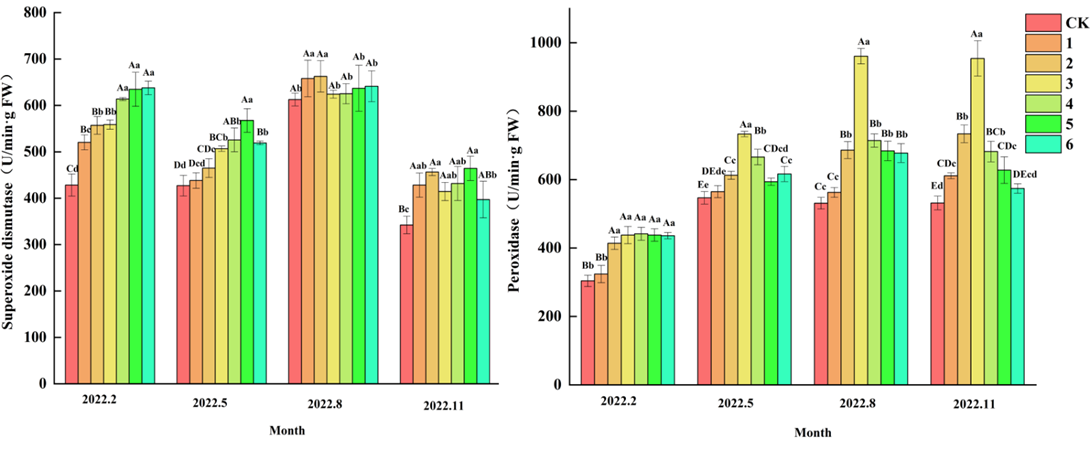
**

Fig 4. Effects of different fertilizer treatments on peroxidase (POD) and superoxide dismutase (SOD) activities of ‘Qi-Nan’ agarwood seedlings.

Raw data for Fig 4 POD

| **Treatment** | **Feb.** | **May.** | **Aug.** | **Nov.** |
| --- | --- | --- | --- | --- |
| ck | 292.98 | 558.654 | 554.26 | 511.56 |
| ck | 295.64 | 556.25 | 515.85 | 542.36 |
| ck | 322.98 | 525.63 | 525.26 | 539.26 |
| 1 | 351.65 | 570.65 | 621.26 | 579.36 |
| 1 | 317.58 | 579.35 | 607.15 | 553.36 |
| 1 | 301.98 | 545.25 | 605.3 | 555.25 |
| 2 | 434.00 | 610.25 | 756.36 | 713.56 |
| 2 | 397.54 | 625.35 | 705.64 | 665.25 |
| 2 | 410.54 | 602.57 | 739.26 | 680.25 |
| 3 | 431.58 | 739.51 | 1003.65 | 952.25 |
| 3 | 416.59 | 724.56 | 900.35 | 943.56 |
| 3 | 465.65 | 735.68 | 958.36 | 986.25 |
| 4 | 461.24 | 689.56 | 716.25 | 736.29 |
| 4 | 423.54 | 664.56 | 670.36 | 700.36 |
| 4 | 440.50 | 644.25 | 659.65 | 706.15 |
| 5 | 455.68 | 605.54 | 639.36 | 684.25 |
| 5 | 439.26 | 584.65 | 584.26 | 712.35 |
| 5 | 419.54 | 591.25 | 659.65 | 655.42 |
| 6 | 438.26 | 642.05 | 586.9 | 708.45 |
| 6 | 444.36 | 600.36 | 559.25 | 669.25 |
| 6 | 426.15 | 607.25 | 576.25 | 655.26 |

**Raw data for Fig 4 POD**

| **Treatment** | **Feb.** | **May.** | **Aug.** | **Nov.** |
| --- | --- | --- | --- | --- |
| ck | 411.84 | 439.54 | 605.82 | 326.03 |
| ck | 417.56 | 401.36 | 603.81 | 363.17 |
| ck | 455.67 | 440.26 | 628.59 | 337.94 |
| 1 | 501.75 | 453.06 | 617.21 | 408.26 |
| 1 | 528.56 | 420.25 | 695.76 | 457.64 |
| 1 | 530.85 | 441.22 | 660.76 | 419.04 |
| 2 | 535.91 | 484.36 | 631.68 | 461.83 |
| 2 | 562.45 | 444.25 | 657.14 | 447.54 |
| 2 | 572.42 | 466.25 | 698.71 | 459.76 |
| 3 | 564.12 | 509.03 | 614.42 | 393.11 |
| 3 | 564.52 | 511.03 | 629.81 | 418.50 |
| 3 | 547.05 | 500.05 | 628.09 | 431.81 |
| 4 | 614.19 | 555.26 | 633.43 | 407.48 |
| 4 | 610.26 | 510.26 | 600.44 | 473.84 |
| 4 | 616.83 | 511.35 | 641.21 | 413.78 |
| 5 | 676.85 | 590.36 | 616.26 | 458.61 |
| 5 | 615.06 | 540.25 | 600.87 | 492.82 |
| 5 | 611.70 | 572.02 | 693.33 | 441.47 |
| 6 | 654.06 | 523.06 | 676.99 | 437.78 |
| 6 | 625.83 | 515.06 | 611.19 | 359.27 |
| 6 | 633.24 | 519.06 | 635.44 | 394.05 |


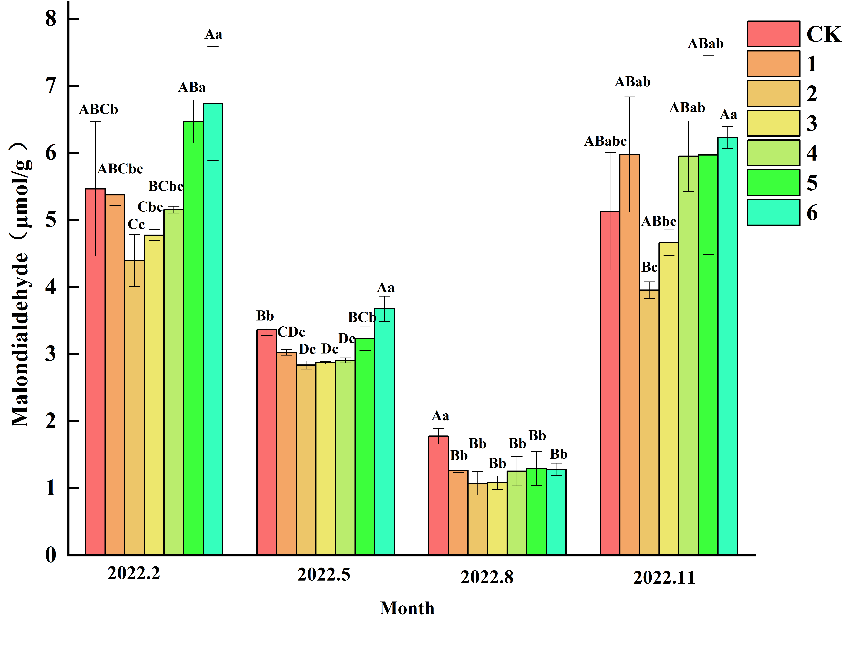


Fig 5. Effects of different fertilizer treatments on the content of malondialdehyde (MDA) in ‘Qi-Nan’ agarwood seedlings.

Raw data for Fig 5

| **Treatment** | **Feb.** | **May.** | **Aug.** | **Nov.** |
| --- | --- | --- | --- | --- |
|  | **（umol/g）** | **（umol/gFW）** | **（umol/gFW）** | **（umol/gFW）** |
| CK | 5.13 | 3.34 | 1.77 | 5.46 |
| 1 | 5.97 | 3.02 | 1.27 | 5.38 |
| 2 | 3.95 | 2.84 | 1.07 | 4.39 |
| 3 | 4.66 | 2.87 | 1.08 | 4.77 |
| 4 | 5.95 | 2.91 | 1.26 | 5.15 |
| 5 | 5.98 | 3.23 | 1.29 | 6.64 |
| 6 | 6.23 | 3.68 | 1.28 | 5.74 |


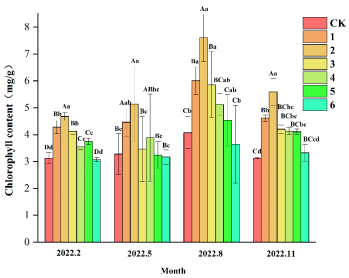


Fig 6. Effects of different fertilization rates on chlorophyll(chl) content of ‘Qi-Nan’ agarwood seedlings.

Raw data for Fig 6

| **Treatment** | **Feb.** | **May.** | **Aug.** | **Nov.** |
| --- | --- | --- | --- | --- |
| ck | 3.07 | 3.28 | 3.91 | 3.25 |
| ck | 3.16 | 2.86 | 2.08 | 3.68 |
| ck | 3 | 3.35 | 4.94 | 3.04 |
| 1 | 2.95 | 2.48 | 4.39 | 3.15 |
| 1 | 3.08 | 3.33 | 3.39 | 3.13 |
| 1 | 3.35 | 4.02 | 4.44 | 3.08 |
| 2 | 4.01 | 4.88 | 6.05 | 4.68 |
| 2 | 4.36 | 3.88 | 5.48 | 4.47 |
| 2 | 4.48 | 4.63 | 6.50 | 4.69 |
| 3 | 4.79 | 4.61 | 6.05 | 5.148 |
| 3 | 4.51 | 6.75 | 5.48 | 5.05 |
| 3 | 4.73 | 4.06 | 11.50 | 6.16 |
| 4 | 4.17 | 2.40 | 7.25 | 4.36 |
| 4 | 4.21 | 4.78 | 4.94 | 4.05 |
| 4 | 4 | 3.20 | 5.38 | 4.21 |
| 5 | 3.47 | 5.45 | 4.71 | 4.18 |
| 5 | 3.65 | 2.22 | 5.11 | 4.23 |
| 5 | 3.51 | 3.99 | 5.53 | 3.99 |
| 6 | 3.84 | 3.77 | 4.84 | 4.22 |
| 6 | 3.61 | 3.19 | 5.30 | 4.02 |
| 6 | 3.79 | 2.76 | 3.46 | 4.08 |


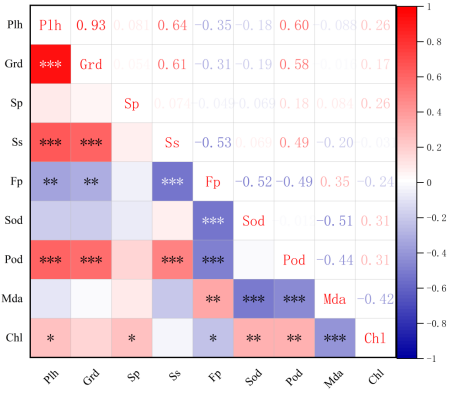


Fig 7. Effects of different fertilization rates on chlorophyll content of agarwood seedlings.

Table 7. Analysis of seedling growth and membership degree of physiological effects of ‘Qi-Nan’ agarwood under different fertilization rates

| **Index** | **CK** | **1** | **2** | **3** | **4** | **5** | **6** |
| --- | --- | --- | --- | --- | --- | --- | --- |
| plant height | 0.00 | 0.51 | 1.00 | 0.69 | 0.34 | 0.51 | 0.34 |
| diameter | 0.10 | 0.34 | 1.00 | 0.68 | 0.41 | 0.33 | 0.00 |
| Soluble protein content | 0.00 | 1.00 | 0.39 | 1.08 | 0.96 | 0.42 | 0.34 |
| Soluble sugar content | 0.00 | 0.26 | 0.40 | 0.72 | 0.54 | 0.60 | 1.00 |
| Free proline content | 1.00 | 0.69 | 0.75 | 0.60 | 0.60 | 0.00 | 0.21 |
| Malondialdehyde content | 0.48 | 0.44 | 0.00 | 0.17 | 0.34 | 1.00 | 0.60 |
| chlorophyll content | 0.00 | 0.63 | 1.00 | 0.47 | 0.41 | 0.42 | 0.15 |
| Superoxide dismutase activity | 0.00 | 0.70 | 0.94 | 0.59 | 0.73 | 1.00 | 0.45 |
| peroxidase activity | 0.00 | 0.12 | 0.48 | 1.00 | 0.36 | 0.23 | 0.10 |
| Mean value of affiliation | 0.18 | 0.52 | 0.66 | 0.67 | 0.52 | 0.50 | 0.35 |
| Order of affiliation | 6 | 3 | 2 | 1 | 3 | 4 | 5 |

**Raw data for Fig 7 and Table 7:All the above data**
