# Supplementary material for: Effects of organic-inorganic complex fertilizer on the growth and physiological characteristics of ‘Qi-Nan’ agarwood from Aquilaria sinensis (Lour.)
Source: PLoS One. 2025 Apr 4;20(4):e0320766. doi: 10.1371/journal.pone.0320766 (PMC11970636; doi:10.1371/journal.pone.0320766)
Supplement: Attachment — Submitted filename: PONE-D-24-45249_reviewer.pdf [file pone.0320766.s002.pdf]

PLOS ONE

Effects of Organic-inorganic Complex Fertilizer on the Growth and Physiological Characteristics of 'Qi-Nan' Agarwood from Aquilaria sinensis (Lour.)

--Manuscript Draft--

**Manuscript Number:**	PONE-D-24-45249
**Article Type:**	Research Article
**Full Title:**	Effects of Organic-inorganic Complex Fertilizer on the Growth and Physiological Characteristics of 'Qi-Nan' Agarwood from Aquilaria sinensis (Lour.)
**Short Title:**	Effects of Organic-inorganic Complex Fertilizer of 'Qi-Nan' Agarwood from Aquilaria sinensis (Lour.)
**Corresponding Author:**	Wei Penglian<br>Guangxi University<br>CHINA
**Keywords:**	'Qi-Nan' agarwood; organic-inorganic complex fertilizer; growth characteristics; physiological characteristics)
**Abstract:**	This paper is to study the effects of different levels of fertilization on the growth, physiological characteristic of 'Qi-Nan' agarwood seedlings. The experiment was conducted under potted conditions using a sin-gle factor experiment. Organic-inorganic complex fertilizer was used as the basal fertilizers, and 7 fertilizer gradients ranging from 0 g/plant to 23 g/plant were set up. The results showed that: (1) within a certain range, with the increase of organic-inorganic complex fertilizer application, the growth and physiological activity of 'Qi-Nan' had an incremental effect, but this effect would be inhibited when the fertilizer application was too high. (2) After fertilization, the contents of free proline (FP) and malondialdehyde (MDA) decreased, while the contents of osmotic regulatory substances and chlorophyll increased. (3) The correlation analysis showed that seedling height and ground diameter of 'Qi-Nan' seed-lings were significantly positively correlated with soluble sugar content, peroxidase activity and chlorophyll content. (4) Membership function analysis showed that organic-inorganic complex fertilizer had a significant effect on the growth of 'Qi-Nan' seedlings, and the optimal fertilizer level of single application of organic-inorganic complex fertilizer was 7-11g/ plant. Therefore, the optimal application level of organic-inorganic complex fertilizer for the seedling cultivation of 'Qi-Nan' was 7g/ plant.
**Order of Authors:**	Wei Penglian
	Jingyue Jingyue
	Yunlin Fu
	Zhu Yu
	Xueting Li
	Siyu Zheng
	Fenyong Tang
**Additional Information:**	
**Question**	**Response**
**Financial Disclosure**<br><br>Enter a financial disclosure statement that describes the sources of funding for the work included in this submission. Review the submission guidelines for detailed requirements. View published research articles from *PLOS ONE* for specific	The author(s) received no specific funding for this work.

Powered by Editorial Manager® and ProduXion Manager® from Aries Systems Corporation

examples.<br><br>This statement is required for submission and **will appear in the published article** if the submission is accepted. Please make sure it is accurate.<br><br>**Funded studies**<br>Enter a statement with the following details:<br>• Initials of the authors who received each award<br>• Grant numbers awarded to each author<br>• The full name of each funder<br>• URL of each funder website<br>• Did the sponsors or funders play any role in the study design, data collection and analysis, decision to publish, or preparation of the manuscript?<br><br>Did you receive funding for this work?	
**Competing Interests**<br><br>Use the instructions below to enter a competing interest statement for this submission. On behalf of all authors, disclose any competing interests that could be perceived to bias this work—acknowledging all financial support and any other relevant financial or non-financial competing interests.<br><br>This statement is required for submission and **will appear in the published article** if the submission is accepted. Please make sure it is accurate and that any funding sources listed in your Funding Information later in the submission form are also declared in your Financial Disclosure statement.<br><br>View published research articles from *PLOS ONE* for specific examples.	The authors have declared that no competing interests exist.

Powered by Editorial Manager® and ProduXion Manager® from Aries Systems Corporation

**NO authors have competing interests**<br><br>Enter: *The authors have declared that no competing interests exist.*<br><br>**Authors with competing interests**<br><br>Enter competing interest details beginning with this statement:<br><br>*I have read the journal's policy and the authors of this manuscript have the following competing interests: [insert competing interests here]*<br><br>* typeset	
**Ethics Statement**<br><br>Enter an ethics statement for this submission. This statement is required if the study involved:<br><br>• Human participants<br>• Human specimens or tissue<br>• Vertebrate animals or cephalopods<br>• Vertebrate embryos or tissues<br>• Field research<br><br>Write "N/A" if the submission does not require an ethics statement.<br><br>General guidance is provided below. Consult the submission guidelines for detailed instructions. **Make sure that all information entered here is included in the Methods section of the manuscript.**	N/A

Powered by Editorial Manager® and ProduXion Manager® from Aries Systems Corporation

**Format for specific study types**<br><br>**Human Subject Research (involving human participants and/or tissue)**<br>• Give the name of the institutional review board or ethics committee that approved the study<br>• Include the approval number and/or a statement indicating approval of this research<br>• Indicate the form of consent obtained (written/oral) or the reason that consent was not obtained (e.g. the data were analyzed anonymously)<br><br>**Animal Research (involving vertebrate animals, embryos or tissues)**<br>• Provide the name of the Institutional Animal Care and Use Committee (IACUC) or other relevant ethics board that reviewed the study protocol, and indicate whether they approved this research or granted a formal waiver of ethical approval<br>• Include an approval number if one was obtained<br>• If the study involved *non-human primates*, add *additional details* about animal welfare and steps taken to ameliorate suffering<br>• If anesthesia, euthanasia, or any kind of animal sacrifice is part of the study, include briefly which substances and/or methods were applied<br><br>**Field Research**<br><br>Include the following details if this study involves the collection of plant, animal, or other materials from a natural setting:<br>• Field permit number<br>• Name of the institution or relevant body that granted permission	
**Data Availability**<br><br>Authors are required to make all data underlying the findings described fully available, without restriction, and from the time of publication. PLOS allows rare exceptions to address legal and ethical concerns. See the PLOS Data Policy and FAQ for detailed information.	Yes - all data are fully available without restriction

A Data Availability Statement describing where the data can be found is required at submission. Your answers to this question constitute the Data Availability Statement and **will be published in the article**, if accepted.<br><br>**Important:** Stating 'data available on request from the author' is not sufficient. If your data are only available upon request, select 'No' for the first question and explain your exceptional situation in the text box.<br><br>Do the authors confirm that all data underlying the findings described in their manuscript are fully available without restriction?	
**Describe where the data may be found in full sentences. If you are copying our sample text, replace any instances of XXX with the appropriate details.**<br><br>• If the data are **held or will be held in a public repository**, include URLs, accession numbers or DOIs. If this information will only be available after acceptance, indicate this by ticking the box below. For example: *All XXX files are available from the XXX database (accession number(s) XXX, XXX.).*<br>• If the data are all contained **within the manuscript and/or Supporting Information files**, enter the following: *All relevant data are within the manuscript and its Supporting Information files.*<br>• If neither of these applies but you are able to provide **details of access elsewhere**, with or without limitations, please do so. For example:<br><br>*Data cannot be shared publicly because of [XXX]. Data are available from the XXX Institutional Data Access / Ethics Committee (contact via XXX) for researchers who meet the criteria for access to confidential data.*<br><br>*The data underlying the results presented in the study are available from (include the name of the third party*	All relevant data are within the manuscript and its Supporting Information files.

and contact information or URL).<br>• This text is appropriate if the data are owned by a third party and authors do not have permission to share the data.<br><br>* typeset	
Additional data availability information:	

Manuscript

Click here to access/download;Manuscript;plos one (2).docx

Effects of Organic-inorganic Complex Fertilizer on the Growth and Physiological Characteristics of 'Qi-Nan' Agarwood from *Aquilaria sinensis* (Lour.)

Jingyue Huang[1], Yunlin Fu[1], Zhu Yu[2], Xueting Li[1], Siyu Zheng[1], Fenyong Tang[2]and Penglian Wei[1*]

[1] Guangxi Colleges and Universities Key Laboratory for Cultivation and Utilization of Subtropical Forest Plantation, College of Forestry, Guangxi University, Nanning 530004, China;
2009392009@st.gxu.edu.cn (J.H.);fuyunlin@gxu.edu.cn (Y.F.); xuetlee@163.com (X.L.); zhengsiyu0416@163.com (S.Z.)
[2] Guangxi Forest Inventory & Planning Institute, Nanning 530011, China;
Yzhu@st.gxu.edu.cn （Z.Y）; tangfenyong83@163.com (F.T)

* Correspondence: weipenglian@gxu.edu.cn; Tel.:+86 18878982397

Abstract: This paper is to study the effects of different levels of fertilization on the growth, physiological characteristic of 'Qi-Nan' agarwood seedlings. The experiment was conducted under potted conditions using a sin-gle factor experiment. Organic-inorganic complex fertilizer was used as the basal fertilizers, and 7 fertilizer gradients ranging from 0 g/plant to 23 g/plant were set up. The results showed that: (1) within a certain range, with the increase of organic-inorganic complex fertilizer application, the growth and physiological activity of 'Qi-Nan' had an incremental effect, but this effect would be inhibited when the fertilizer application was too high. (2) After fertilization, the contents of free proline (FP) and malondialdehyde (MDA) decreased, while the contents of osmotic regulatory substances and chlorophyll increased. (3) The correlation analysis showed that seedling height and ground diameter of 'Qi-Nan' seed-lings were significantly positively correlated with soluble sugar content, peroxidase activity and chlorophyll content. (4) Membership function analysis showed that organic-inorganic complex fertilizer had a significant effect on the growth of 'Qi-Nan' seedlings, and the optimal fertilizer level of single application of organic-inorganic complex fertilizer was 7-11g/ plant. Therefore, the optimal application level of organic-inorganic complex fertilizer for the seedling cultivation of 'Qi-Nan' was 7g/ plant.

Keywords: 'Qi-Nan' agarwood; organic-inorganic complex fertilizer; growth characteristics; physiological characteristics)

1. Introduction

Agarwood (Chen-Xiang in Chinese), the resinous heartwood from the Aquilaria species (Thymelaeaceae) [1], has widely used in medicine, aromatherapy, perfume industry, religious culture, and other fields. It is a valuable traditional medicine and natural spice [2-4]. 'Qi-Nan', also known as Kanankoh, Kyara [5,6] or Chi-Nan [7]. The wild 'Qi-Nan' is usually considered to be the highest quality wild agarwood wood, and its price is thousands of times higher than ordinary agarwood [8]. However, due to the destruction of the natural environment and excessive deforestation caused by economic interests, wild *Aquilaria* species are endangered [9]. This genus is now listed in Appendix II of the Convention on Internal Trade in Endangered Species of Wild Fauna and Flora [10] Additionally, the low yield of wild Aquilaria species cannot meet the market demand. Therefore, planting agarwood trees has become an important way to obtain agarwood. In recent years, grafted 'Qi-Nan' agarwood tree has emerged. It is a high-quality agarwood obtained by grafting and breeding from natural wild agarwood trees [11,12]. *Aquilaria sinensis* "Reke2" is an example of high-quality cultivated "Qi-Nan" propagated by grafting with the ordinary germplasm of *Aquilaria sinensis* [13] It has been

cultivated in places such as Hainan, Guangdong, Guangxi, and Yunnan, with excellent results. Compared to trees grown from ordinary agarwood seedlings, grafted "Qi-Nan" agarwood can more easily produce higher-quality agarwood and has the advantages of early incense-bearing and high yield [11, 14]. In order to satisfy the demand of the agarwood market, 'Qi-Nan' agarwood are being vigorously promoted and planted in China. According to preliminary statistics, 5,766 acres of 'Qi-Nan' agarwood seedlings have been planted nationwide [15-17]. Fertilization is a key measure for seedling planting [18] and is essential for the healthy growth of trees, which is the basis for forming more agarwood.

China's traditional crops and forestry on the planting of chemical fertilizer used in a single species, organic fertilizer accounted for a low proportion of fertilizer nutrient distribution ratio unreasonable is an important reason for the low utilization of fertilizer, and over-fertilization will cause a series of environmental pollution problems [19]. Some studies have pointed out that under the same nutrient content conditions, the nutritional effect of organic fertilizers applied in combination with chemical fertilizers exceeds that of single application of chemical fertilizers or single application of organic fertilizers, and at the same time, it plays a role in promoting the mineralization of organic fertilizers, activating the phosphorus in the soil, reducing the fixation of inorganic phosphorus, and extending the fertilizer supplying performance of chemical nitrogen fertilizers [20]. The application of organic-inorganic complex fertilizer is mostly seen in crops [21-24], and its application has reached a certain scale, and the related research has also reached a certain degree. However, the application of organic-inorganic fertilizers on forest trees is relatively small, only focusing on the application of common tree species, such as *Cinnamomum camphora* [25], *Larix gmelinii* [26], *Eucalyptus* [27], etc. The application of organic-inorganic complex fertilizer on agarwood is rarely researched, especially on such precious tree species as 'Qi-Nan' agarwood.

At the fertilization level of agarwood plants, some scholars have studied the fertilization methods of agarwood. For example, the combination application of 10 g biological bacterial fertilizer and 20 g complex fertilizer has the best effect on promoting the growth of *Aquilaria sinensis* seedlings and enhancing soil fertility [28]. The use of a combination of inorganic fertilizer, organic fertilizer and biofertilizer treatments can better promote the growth of agarwood seedlings [29]. In addition, the fertilization method of *Aquilaria malaccensis* was also preliminarily explored. The photosynthetic capacity, growth amount and biomass of *Aquilaria malaccensis* were maximized when N was applied at 3 000 mg/plant. And when N was applied at 3.637g/ plant, P was applied at 2.5g/ plant and K was applied at 1.25g/ plant, the maximum biomass of *Aquilaria malaccensis* could be obtained at 27.688 g [29]. At present, however, there is a lack of studies on the fertilization and growth of 'Qi-Nan' seedlings. For example, the effects of different fertilization level on the growth and physiology of grafted 'Qi-Nan' agarwood seedlings have not been reported. The background that the quality and yield of 'Qi-Nan' agarwood are far from meeting the market demand, how to adopt a reasonable fertilization mode to achieve efficient seedling breeding is a significant problem; this problem needs to be solved urgently in the current cultivation process of "Qi-Nan" agarwood seedlings.

Therefore, in this study, organic-inorganic complex fertilizer was used as base fertilizers to study the effects on the growth and physiological characteristics of 'Qi-Nan' agarwood seedlings. The purpose of this study was threefold: (1) to determine the changes of the growth and physiology of 'Qi-Nan' seedlings under different fertilizing amounts; (2) to determine which physiological and nutrient indexes were the key factors affecting the height and ground diameter of 'Qi-Nan' seedlings; (3) to explore the level of organic-inorganic complex fertilizer application that is most conducive to the growth and development of 'Qi-Nan' agarwood seedlings

2. Materials and Methods

2.1 Experimental Materials

The experimental varieties were obtained from the seedling breeding base in Nalou Town, Nanning, Guangxi (108°63 'E, 22°60 'N), which has a humid subtropical monsoon climate, with an average annual rainfall of 1304.2 mm, an average annual temperature of 21.6 °C and an average annual relative humidity of 79 % [31]. Grafted 'Qi-Nan' agarwood seedlings with healthy and consistent growth for 2 months were selected as experimental materials in October 2021. The average seedling height was 17.60 ± 0.33 cm and the average ground diameter was 4.95 ± 0.29 mm. The roots of the seedlings were kept clean before transplanting and transplanted into a plastic pot (with a tray) with a diameter of 264 mm and a height of 239 mm. The local yellow loam and sandy soil were mixed 1:1 as potting soil, and each pot was filled with 0.5 kg soil. The background values of the soil were measured before the experiment. The pH was 4.07, the alkali-hydrolyzed nitrogen was 29.43 mg/kg, the available potassium was 77.69 mg/kg, the available phosphorus was 30.14 mg/kg, and the organic matter content was 17.1 g/kg. The fertilizer used in the test was potassium sulfate compound fertilizer, produced by Miaoran Agricultural Chemical Technology Co., LTD., with total nitrogen ≥ 15%, total potassium ≥ 15%, total phosphorus ≥ 15%, and total nutrient content ≥ 45%.

2.2 Experiment Design

The experiment was a single factor experiment of organic-inorganic complex fertilizer. 7 levels of organic-inorganic complex fertilizer were set to fertilize the current year's sown seedlings of 'Qi-Nan' agarwood. A total of 7 treatments were set up, CK (0 g/ plant), 1 (3 g/ plant), 2 (7 g/ plant), 3 (11 g/ plant), 4 (15 g/ plant), 5 (19 g/ plant), 6 (23 g/ plant). Each treatment was 12 plants with 3 replications. After a month of rejuvenation period, the grafted ‘Qi-Nan’ agarwood seedlings were fertilized. The background values of seedling height and ground diameter were measured before fertilization. All fertilization treatments were completely consistent in the management of supply, weeding, pest control and so on during the experiment, to minimize the experimental errors in the process of fertilization treatment. Fertilizer was applied twice, in November 2021 and May 2022, respectively, because of the seasonal nutrient uptake pattern of seedlings considered in the pot experiment. Evenly sprinkle the corresponding amount of organic and inorganic complex fertilizer on the surface of the potting soil, and then water when fertilizing. The experiment was conducted from November 2021 to November 2022, and the experimental period was one year.

2.3 Sample Collection and Determination

2.3.1 Determination of Growth Index

The plant height and ground diameter of ‘Qi-Nan’ agarwood seedlings were measured with steel tape (accuracy of 0.1cm) and vernier caliper (accuracy of 0.1mm), respectively. The measurements were taken from November 2021 to November 2022, and the heights and ground diameters of all potted seedlings were measured every other month for a total of 12 times, each at the exact location.

2.3.2 Measurement of Physiological Indexes and Leaf Nutrient Content

For each treatment, three well-grown seedlings were randomly selected, and the upper branches were used to count the 2nd to 5th mature functional leaves from the terminal bud downwards. These leaves were wiped clean, put into plastic bags, labeled, and temporarily stored in ice boxes; after collection, they were brought back to the laboratory and stored in an ultra-low-temperature refrigerator (-70 ◦C). The contents of chlorophyll, soluble sugar, soluble protein, free proline, and malondialdehyde were determined by the acetone extraction method [32], anthrone colorimetric method [33], Coomassie brilliant blue method [34], acid ninhydrin colorimetric method [35], and thiobarbituric acid colorimetric method [33], respectively. The activities of superoxide dismutase and peroxidase were determined by acid ninhydrin colorimetry [36] and guaiacol colorimetry [33]. The measurement time was February 2022, May 2022, August 2022, and November 2022, with a total of 4 measurements.

2.3.3 Data Analysis and Processing Methods

The data were organized by Excel 2016; The SPSS 25.0 software (SPSS Inc., Chicago, IL, USA) was used to perform the statistical analyses by ANOVA and significance tests. p ≤ 0.05 was considered to indicate statistically significant differences. Plotting was performed by origin 2021; The membership function method of fuzzy mathematics was used for the comprehensive evaluation of the growth and physiological indexes of 'Qi-Nan' agarwood under the effect of organic-inorganic complex fertilizer.

3. Results

3.1 Effects of Organic-Inorganic Complex Fertilizer on Growth Indexes of 'Qi-Nan' Agarwood Seedlings

3.1.1. Effects of Organic-Inorganic Complex Fertilizer on Seedling Height of 'Qi-Nan' Agarwood Seedlings

From figure 1 that under different fertilization treatments, the dynamic change of plant height growth of 'Qi-Nan' agarwood seedlings showed an upward trend, and the growth rhythm was characterized by "slow-fast-slow" and showed an S-shaped curve. After May of the following year, the seedlings entered the rapid growth period, the seedling height growth increased significantly, in which the height growth of the seedlings was extremely rapid from June to August, and they entered the peak period of seedling height growth.

From table.1, the differences in seedling height between groups of 'Qi-Nan' agarwood seedlings at the end of fertilization were significant ($P < 0.05$). The results of multivariate analysis of variance showed that different amounts of organic-inorganic complex fertilizer treatment and different growth months had significant effects on plant height growth ($P < 0.01$). Among them, the seedling height and total growth of 'Qi-Nan' agarwood seedlings were the largest in treat 2, 81.8 cm and 64.7cm, respectively, which were 144.5% and 167.3% of CK, followed by treat 3. The seedling height and total growth of 'Qi-Nan' agarwood in treat 4 were the smallest, 65.1cm and 47.3cm, respectively, which were 115.0% and 122.5% of CK.

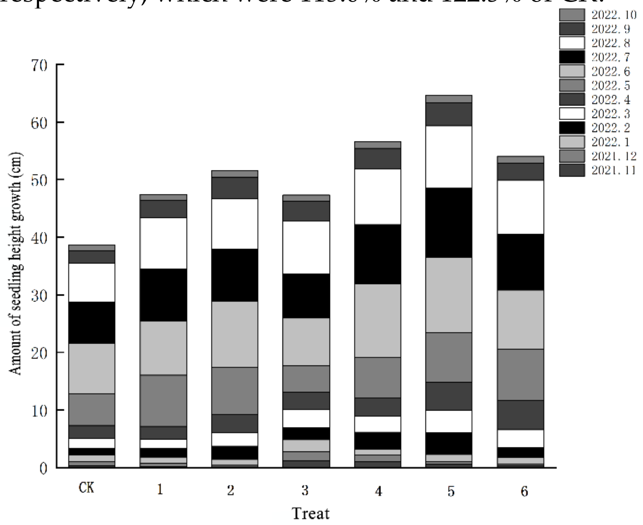

Figure 1. Variation in seedling height growth of 'Qi-Nan' agarwood seedlings

Table 1. Dynamics of seedling height growth of 'Qi-Nan' agarwood seedlings under different fertilization rates (Units: cm)

**Month**	**Treatment**						
	**CK**	**1**	**2**	**3**	**4**	**5**	**6**
Nov.	17.9	17.2	17.1	17.5	17.8	18.0	17.7
Dec.	18.2	17.6	17.7	18.5	18.9	18.1	17.9
Jan.	18.9	17.9	18.1	19.6	20.5	18.4	18.4
Feb.	20.0	18.9	19.4	20.7	22.6	19.4	19.5
Mar.	21.3	20.7	23.1	23.6	24.6	21.7	21.1
Apr.	22.9	23.8	27.1	26.4	27.9	24.0	22.6
May.	25.2	28.9	32.0	29.6	30.9	27.3	24.9
Jun.	30.7	37.8	40.6	36.6	35.4	35.4	33.8
Jul.	39.5	46.3	53.7	49.4	43.7	46.9	43.2
Aug.	46.7	56.0	65.7	59.7	51.4	56.0	52.2
Sep.	53.5	65.4	76.5	69.3	60.6	64.7	61.1
Oct.	55.6	68.4	80.6	72.9	64.0	68.4	64.1
Nov.	56.6	69.6	81.8	74.1	65.1	69.5	65.2
Total increment	38.7±3.2De	52.4±4.9Cbc	64.7±5.6Aa	56.6±3.2Bb	47.3±4.2Ccd	51.5±5.0Cbd	47.5±3.8Ccd
Relative increment	100.0%	135.4%	167.3%	146.4%	122.5%	133.3%	122.7%

ANOVA
e.g.<0.001
<0.05
r(p)

Note: The data in the table are mean ± standard deviation; Different capital letters indicate highly significant differences between the same index, the same period, and different treatments at the 0.01 level ($P < 0.01$), and different lowercase letters indicate significant differences between the same index, the same period, and different treatments at the 0.05 level ($P < 0.05$).

3.1.2 Effect of Organic-Inorganic Complex Fertilizer on the Ground Diameter of 'Qi-Nan' Agarwood Seedlings

From figure 2 that in the early stage of fertilization (2021.11-2022.2), the effect of each treatment on the growth of 'Qi-Nan' incense was not obvious, and the growth of seedling diameter was still slow in March and April. From May to June is the peak period of the growth of the ground diameter, and the growth of the ground diameter in all treatments is larger in June. After June, the growth rate decreased, and the increase in diameter of each treatment was more average in different months from June to October.

From table.2, after fertilization, the seedling height and ground diameter of 'Qi-Nan' agarwood seedlings had significant differences among groups ($P < 0.05$). The results of multivariate analysis of variance showed that different amounts of organic-inorganic complex fertilizer treatment and different growth months on plant height growth were highly significant ($P < 0.01$). Among them, treat 2 had the largest growth in diameter and total diameter, which were 160.38% and 214.24% of CK, followed by treat 3. Treat 6 had the smallest growth in diameter and total diameter, which were 0.83 mm and 0.33 mm less than that of the CK, and some of the seedlings in treat 6 were dead, which may be due to 'burnt' caused by the application of the high-concentration fertilizers.

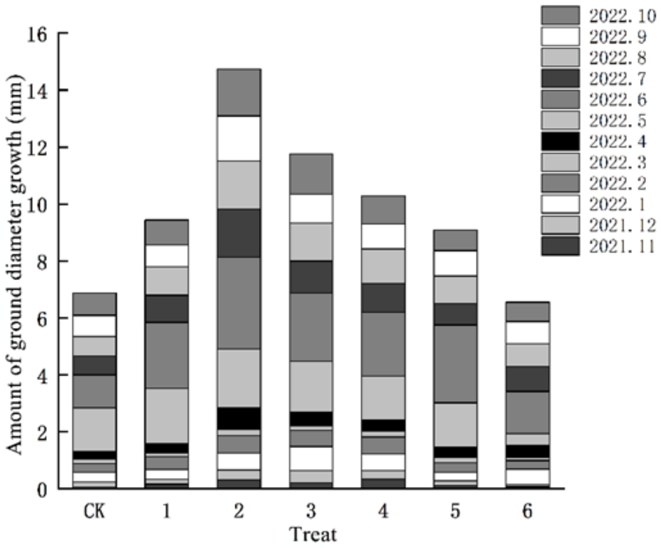

Figure 2. Variation in ground diameter growth of 'Qi-Nan' agarwood seedling

Table.2 Dynamics of ground diameter growth of 'Qi-Nan' agarwood seedlings under different fertilization rates (Units: mm)

**Month**	**Treatment**						
	**CK**	**1**	**2**	**3**	**4**	**5**	**6**
Nov.	5.36	4.76	4.89	5.26	4.53	5.01	4.86
Dec.	5.42	4.92	5.20	5.47	4.87	5.13	4.93
Jan.	5.60	5.09	5.55	5.90	5.17	5.29	5.01
Feb.	5.94	5.44	6.15	6.74	5.75	5.59	5.55
Mar.	6.25	5.89	6.76	7.31	6.35	5.93	5.85
Apr.	6.40	6.02	6.99	7.48	6.55	6.11	5.97
May.	6.66	6.35	7.74	7.94	6.95	6.47	6.38
Jun.	8.20	8.28	9.79	9.74	8.48	8.03	6.80
Jul.	9.36	10.60	13.03	12.13	10.73	10.76	8.28
Aug.	10.02	11.56	14.71	13.26	11.74	11.52	9.16
Sep.	10.71	12.55	16.40	14.59	12.95	12.48	9.95
Oct.	11.45	13.32	17.98	15.61	13.82	13.37	10.73
Nov.	12.24	14.20	19.63	17.02	14.81	14.10	11.41
Total increment	6.88±0.89d	9.44±1.01c	14.74±1.50a	11.76±1.24b	10.28±1.10bc	9.09±0.76c	6.55±0.75d
Relative increment	100.00%	137.21%	214.24%	170.93%	149.42%	132.12%	95.20%

Note: The data in the table are mean ± standard deviation; Different capital letters indicate highly significant differences between the same index, the same period, and different treatments at the 0.01 level ($P < 0.01$), and different lowercase letters indicate significant differences between the same index, the same period, and different treatments at the 0.05 level ($P < 0.05$).

3.2 Effects of Organic-Inorganic Complex Fertilizer on Physiological and Biochemical Indexes of 'Qi-Nan' Agarwood Seedlings

3.2.1 Effects of Organic-Inorganic Complex Fertilizer on the Content of Osmotic Mediating Substances in 'Qi-Nan' Agarwood Seedlings

According to the results of the effects on soluble protein (SP) (Figure.3a), the SP content of 'Qi-Nan' agarwood seedlings differed between groups ($P < 0.05$) in February, May, August and November. The results of multivariate analysis of variance showed that the effects of different fertilizer amount of organic-inorganic complex fertilizer treatments and different growth months on SP content were highly significant ($P < 0.01$). Overall, the SP content of 'Qi-Nan' agarwood seedlings did not change significantly

over time under different amounts of organic and inorganic compound fertilizer. With the increase of fertilizer application, the SP content generally increased first and then decreased. Except that the SP content of treat 2 in November was lower than that of treat 3, the SP content of treat 2 and treat 3 in other months was higher, indicating that the two concentration levels of fertilization had a better effect on the synthesis of SP content in seedlings. Within a certain range, the SP content of 'Qi-Nan' agarwood seedlings also increased with the increase of the amount of organic-inorganic complex fertilizer application, but the SP content would decrease beyond a certain concentration.

According to the results of the effect on soluble sugar (SS) (Figure.3b), the SS content of 'Qi-Nan' agarwood seedlings differed between groups ($P < 0.05$) in February, May, August and November. The results of multivariate analysis of variance showed that the effects of different amounts of organic-inorganic compound fertilizer treatment and different growth months on SS content were highly significant ($P < 0.01$). Overall, the SS content of 'Qi-Nan' agarwood seedlings showed an increasing trend over time, and the overall change was small. The SS content in February increased first and then decreased with the increase of fertilizer application. In August, the SS content of treat 3 and 4 was lower than that of CK, but entered November, the SS content showed an upward trend with the increase of fertilizer application, and the SS content of each fertilizer treatment was higher than that of CK, indicating that organic and inorganic compound fertilizer had a better promotion effect on the synthesis of SS. Meanwhile, the SS content of treat 6 reached the maximum.

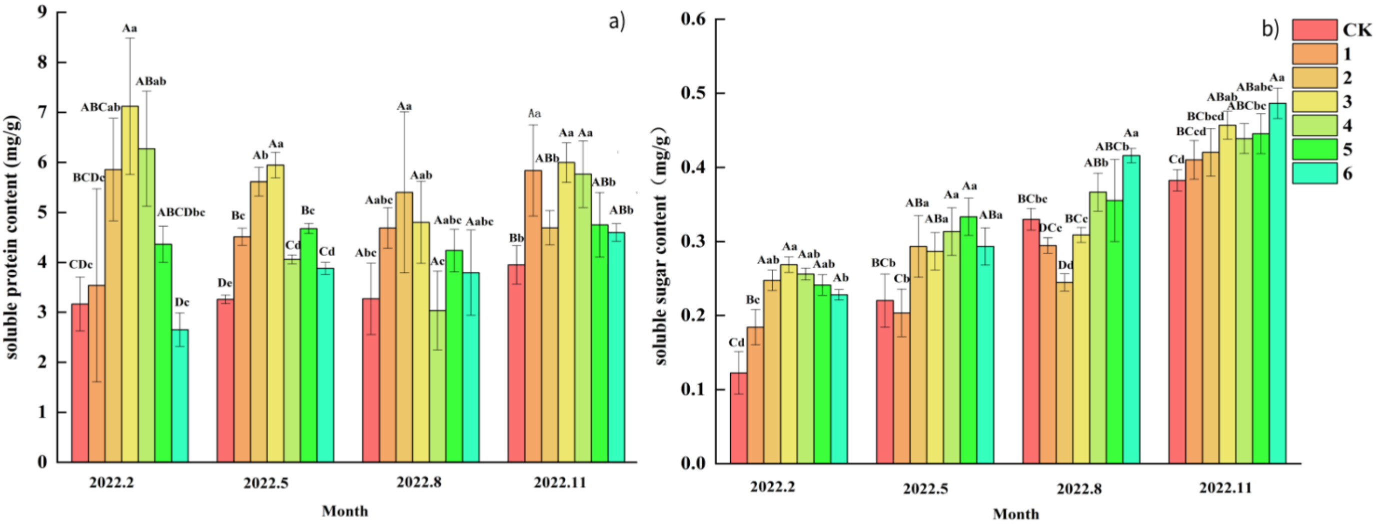

Figure.3 Effects of different fertilizer treatments on soluble protein (SP) and soluble sugar (SS) content of 'Qi-Nan' agarwood seedlings. **(a)** soluble protein; **(b)** soluble sugar. Different capital letters indicate highly significant differences between the same index, the same period, and different treatments at the 0.01 level ($P < 0.01$), and different lowercase letters indicate significant differences between the same index, the same period, and different treatments at the 0.05 level ($P < 0.05$).

3.2.2 Effect of Bio-Organic Fertilizer Treatments on Enzyme Activity of "Qi-Nan" Agarwood Seedlings

From the results of the effect on peroxidase (POD) activity (Figure. 4a), there were differences ($P < 0.05$) between groups of POD activity in 'Qi-Nan' seedlings in February, May, August and November. The results of multifactorial ANOVA showed that the effects of different fertilizer doses of organic and inorganic composite fertilizer treatments and different growth months on POD activity were highly significant ($P < 0.01$). Under different fertilization treatments of organic and inorganic compound fertilizers, the POD activity of 'Qi-Nan' seedlings showed an increasing and then decreasing trend with the increase of fertilization. Throughout the growth process, POD activity showed a general upward trend with time, indicating that peroxidase activity

was weak in young seedlings and high in mature seedlings. POD activity was higher in all fertilization treatments than in CK. During the month of November, the POD activity of treatments 1, 2, and 3 increased compared to August, whereas treatments 4, 5, and 6 decreased compared to August, indicating that the POD activity continued to increase at low concentrations, and was higher in the early part of the year and lower in the later part of the year at high concentrations.

From the results of the effect on superoxide dismutase (SOD) activity (Figure. 4b), there were differences (P < 0.05) between groups of SOD activity in 'Qi-Nan' seedlings in February, May, August and November. The results of multifactorial ANOVA showed that the effects of different fertilizer application rates of organic-inorganic composite fertilizer treatments and different growing months on SOD activity were highly significant (P < 0.01). The SOD activity of 'Qi-Nan' seedlings increased with the increase of organic and inorganic composite fertilizers in each fertilization treatment. In February and May, the SOD activity of treatment 6 was high compared to that of treatments CK, 1, 2, and 3 with low concentration, while in November it was low compared to that of treatments with low concentration, indicating that superoxide dismutase activity was higher in the early stage of the treatment with high fertilizer concentration, and declined or flat in the later stage of the treatment; the SOD activities of the control CK and the treatments 1, 2, and 3 with low concentration largely showed an increase with the increase of the concentration throughout the growth process, and the SOD activeness could be maintained with a sustained growth in the low concentration.

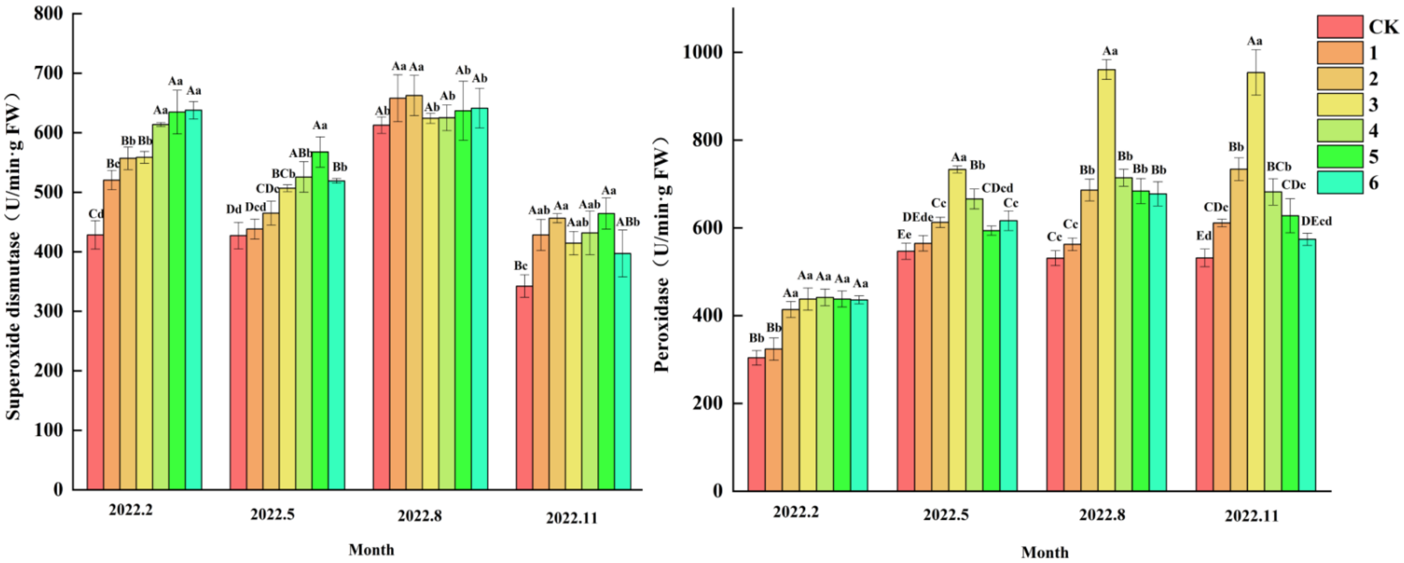

Figure 4 Effects of different fertilizer treatments on peroxidase (POD) and superoxide dismutase (SOD) activities of 'Qi-Nan' agarwood seedlings. **(a)** peroxidase; **(b)** superoxide dismutase. Different capital letters indicate highly significant differences between the same index, the same period, and different treatments at the 0.01 level (P < 0.01), and different lowercase letters indicate significant differences between the same index, the same period, and different treatments at the 0.05 level (P < 0.05).

3.2.3 Effect of Fertilization on Malondialdehyde in 'Qi-Nan' Seedlings

From the results of the effect on malondialdehyde (MDA) (Figure. 5), there were differences (P < 0.05) between groups in the MDA content of 'Qi-Nan' seedlings in February, May, August and November. The results of multifactorial ANOVA showed that the effects of different fertilizer application rates of organic-inorganic composite fertilizer treatments and different growing months on MDA content were highly significant (P < 0.01). The MDA content of 'Qi-Nan' seedlings varied greatly among different fertilizer treatments and different months throughout the growth process. The changes in MDA content of 'Qi-Nan' seedlings treated with different fertilization rates in different months were basically the same, generally showing a decreasing and then

increasing trend. The MDA content of the treatments was comparable in February and November, both of which were significantly higher than in May and August, while the lowest MDA content was found in all treatments in August.

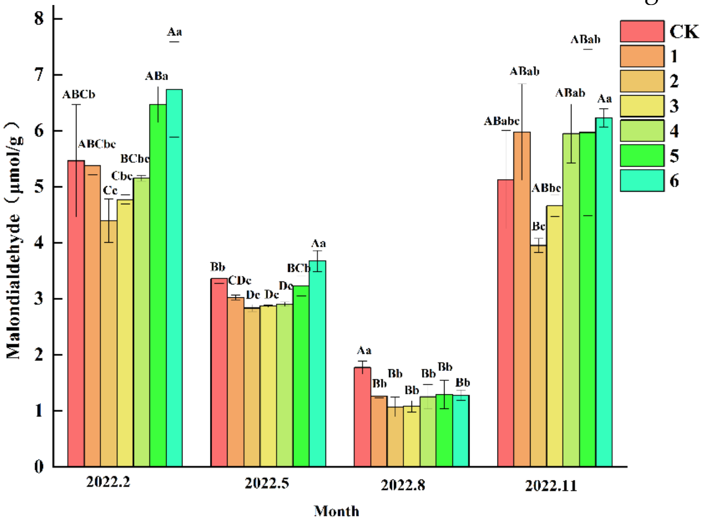

Figure 5. Effects of different fertilizer treatments on the content of malondialdehyde (MDA) in ‘Qi-Nan’ Agarwood seedlings. Different capital letters indicate highly significant differences between the same index, the same period, and different treatments at the 0.01 level ($P < 0.01$), and different lowercase letters indicate significant differences between the same index, the same period, and different treatments at the 0.05 level ($P < 0.05$).

3.2.4 Effects of Fertilization on Chlorophyll Content of ‘Qi-Nan’ Seedlings

From the results of the effect on chlorophyll (Chl) content (Figure. 6), the Chl content of ‘Qi-Nan’ seedlings differed between groups ($P < 0.05$) in February, May, August and November. The results of multifactorial ANOVA showed that the effects of different fertilizer rates of organic-inorganic composite fertilizer treatments and different growing months on Chl content were highly significant ($P < 0.01$). Overall, it appeared that the Chl content of ‘Qi-Nan’ seedlings increased and then decreased with the time of fertilization, reaching a maximum in August, when photosynthesis was strongest. Except for treatment 6, the Chl content of all treatments was higher than that of CK, indicating that organic and inorganic composite fertilizers can effectively promote the growth of Chl within a certain range of fertilizer application. The Chl content of treatment 6 was lower than that of CK in the periods of February, May, and August, and slightly higher than that of CK in November, indicating that the application of high concentration of organic and inorganic compound fertilizer in the early stage exerted a stressful effect on the ‘Qi-Nan’ sedum seedling, which inhibited Chl synthesis in the seedling. As the root system of the seedlings grew later in the season, the seedlings became more resilient, allowing the chlorophyll content to be gradually higher than that of CK.

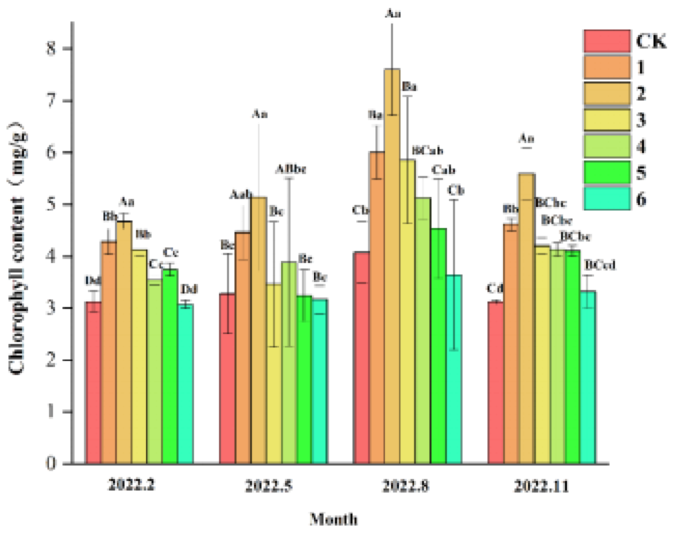

Figure 6. Effects of different fertilization rates on chlorophyll(chl) content of Agarwood sinensis seedlings. Different capital letters indicate highly significant differences between the same index, the same period, and different treatments at the 0.01 level (P < 0.01), and different lowercase letters indicate significant differences between the same index, the same period, and different treatments at the 0.05 level (P < 0.05).

4 Comprehensive Analysis of the Indicators of 'Qi-Nan' Seedlings

Correlation analysis of different growth and physiological indices in each month showed that seedling height was highly significantly positively correlated with SS and POD, and significantly positively correlated with Chl; Ground diameter was highly significantly and positively correlated with SS, Chl and POD; POD showed highly significant positive correlation with SS, significant positive correlation with Chl and significant negative correlation with MDA; MDA showed highly significant negative correlation with Chl and SOD. This indicates that when the Chl content, SS content and POD activity are higher, the seedling height and diameter of 'Qi-Nan' seedlings will grow better; on the contrary, when the Chl content, SS content and POD activity are lower and the MDA content is higher, the seedling height and diameter of 'Qi-Nan' seedlings will grow worse.

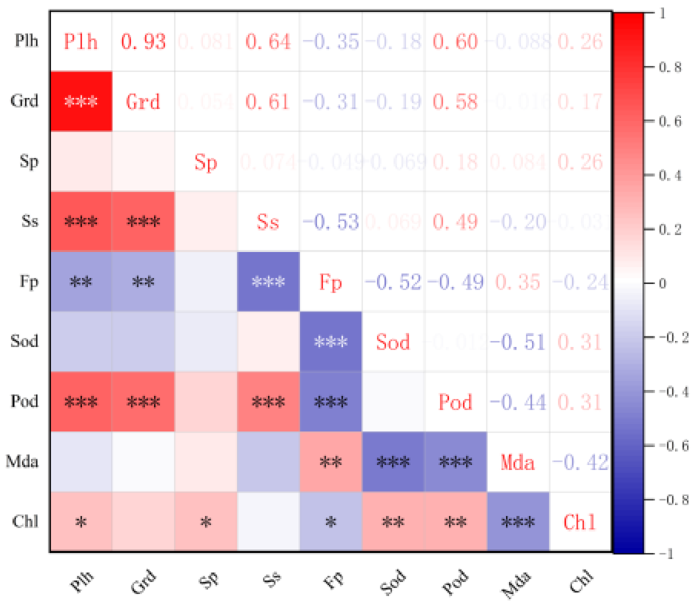

Figure 7. Effects of different fertilization rates on chlorophyll content of Agarwood seedlings. **(a)** plant height (PlH); **(b)** ground diameter (GrD); **(c)** soluble protein (SP) **(d)** soluble sugar (SS); **(e)** Free proline (FP); **(f)** superoxide dismutase (SOD); **(g)** peroxidase (POD); **(h)** malondialdehyde (MDA); **(i)** chlorophyll (Chl). p<0.5: *; p<0.1: **; p<0.01: ***

Based on the results of the above growth and physiological condition tests, membership function analysis was used to synthesize and analyze the results in Table 3. Based on the mean value of affiliation, it can be concluded that treatment 3 has the highest composite score, followed by treatment 2, treatment 5 and treatment 4. The composite ranking is 3 > 2 > 5 > 4 > 1 > 6 > CK.

Among them, the score of treatment 5 is higher than that of treatment 4, which may be due to the fact that the malondialdehyde score of treatment 5 is much larger than that of treatment 4, while the scores of the other indicators do not differ much, which makes the mean value of the affiliation of each indicator of treatment 5 larger than that of treatment 4.It showed that treatment 5 was subjected to greater stress and produced more malondialdehyde than treatment 4; treatments 4 and 5 scored higher than treatments 1 and 6, indicating that treatment 6 was over-fertilized compared to treatments 4 and 5, and that treatment 1 was under-fertilized compared to treatments 4 and 5, and that over- or under-fertilizing was detrimental to the growth of seedlings; and treatment 1 scored higher than treatment 6, suggesting that organic and inorganic composite fertilizers at high concentrations inhibited the growth of seedlings.

Table 3. Analysis of seedling growth and membership degree of physiological effects of 'Qi-Nan' Agarwood under different fertilization rates

Index	CK	1	2	3	4	5	6
plant height	0.00	0.51	1.00	0.69	0.34	0.51	0.34
diameter	0.10	0.34	1.00	0.68	0.41	0.33	0.00
Soluble protein content	0.00	1.00	0.39	1.08	0.96	0.42	0.34
Soluble sugar content	0.00	0.26	0.40	0.72	0.54	0.60	1.00
Free proline content	1.00	0.69	0.75	0.60	0.60	0.00	0.21
Malondialdehyde content	0.48	0.44	0.00	0.17	0.34	1.00	0.60
chlorophyll content	0.00	0.63	1.00	0.47	0.41	0.42	0.15
Superoxide dismutase activity	0.00	0.70	0.94	0.59	0.73	1.00	0.45
peroxidase activity	0.00	0.12	0.48	1.00	0.36	0.23	0.10
Mean value of affiliation	0.18	0.52	0.66	0.67	0.52	0.50	0.35
Order of affiliation	6	3	2	1	3	4	5

5 Discussion

5.1 Effects of Organic and Inorganic Compound Fertilizer on Growth Indexes of Agarwood Seedlings of 'Qi-Nan'

Plant height, ground diameter and so on, as the key growth indicators of forest growth, can well reflect the growth state of forest [37]. The seedling height and diameter were higher than CK in all fertilizer treatment groups in this experiment, except for treatment 6, which had a slightly smaller diameter than CK. Among them, the seedling height and ground diameter of treatment 2 reached the maximum among the groups, which were 44.52% and 60.38% higher than CK, respectively. This suggests that moderate application of organic-inorganic complex fertilizers will promote the growth of 'Qi-Nan' seedlings, which is similar to the findings of olive tree [38], *Aquilaria sinensis* [29], *Aquilaria yunnanensis*[39] and *Corylus heterophylla* [40].However, fertilization should be appropriate, too high or too low fertilization are not conducive to the growth of seedlings, too low fertilization can not provide seedlings with sufficient nutrients, while too high fertilization may cause seedlings of the "seedling burning" phenomenon, the seedlings have a toxic effect. In this experiment, under the treatment of 7-11g/plant of organic-inorganic complex fertilizer, the growth performance of 'Qi-Nan' seedlings was optimal, and the growth condition of the treatment group with more than 11g/plant became worse gradually with the increase of fertilizer application. This indicates that within a certain range of fertilizer application, a reasonable amount of fertilizer will significantly promote seedling growth, while too high or too low fertilizer application will be detrimental to seedling's growth. This is consistent with the results of the study of *Sapindus mukorossi* [41] and *Amorpha fruticose* [42] seedlings. With the increase of fertilizer application, the growth indicators of *Sapindus mukorossi* and *Amorpha fruticose* showed a tendency of increasing and then decreasing. In addition, the growth trend of seedlings in the whole stage is "slow, fast and slow", which may be attributed to the weak root system of seedlings in the early stage of growth, the application of organic-inorganic complex fertilizers and the cold weather in winter produced a certain degree of stress on the plant, which requires a period of slowing down the seedling period.

5.2 Effects of Organic and Inorganic Fertilizers on the Physiology of 'Qi-Nan' Seedlings

The effects of different concentrations of organic-inorganic complex fertilizers on the physiological indexes of 'Qi-Nan' seedlings varied. In this experiment, the chlorophyll content, soluble protein content, and POD content of seedlings showed a trend of increasing and then decreasing with the increase in the amount of fertilizer, which is consistent with the results of different nitrogen, phosphorus, and potassium application on buckwheat leaves [43], and different concentrations of water-fertilizer coupling on greenhouse bag-grown tomatoes [44].This suggests that a reasonable amount of fertilizer will improve the photosynthetic capacity of 'Qi-Nan' seedlings and promote plant carbon and nitrogen metabolism. SS and SP are both indispensable nutrients in plants, and they also play an important role in maintaining osmoregulation in plants [45]. When the concentration of organic-inorganic complex fertilizers was too high, the SS content kept increasing, indicating that the high concentration of organic-inorganic complex fertilizers promoted the SS content of 'Qi-Nan' seedlings better, while the content of SP appeared to be decreasing instead. The reason may be that the high concentration of fertilizer caused high protease activity in 'Qi-Nan' seedlings, which accelerated the rate of protein hydrolysis, while RNA transcription and translation were inhibited [46], resulting in a decrease in the SP content in 'Qi-Nan' seedlings. This is in line with the results emerging from the study of physiological and ecological adaptation of *Suaeda heteropptera Kitagawa* to salt stress alleviation [47]. FP and MDA are indicators of stress tolerance in plants [48], and with the increase of o organic-inorganic complex fertilizer application, the content of MDA and FP first decreased and then increased, indicating that the appropriate concentration of organic-inorganic complex fertilizer can reduce the environmental stress suffered by 'Qi-Nan' seedlings. Under adverse conditions, the metabolism of reactive oxygen species in plants is imbalanced with the scavenging of the defense system and the membrane structure is disrupted [49].In contrast, POD and SOD, as protective enzyme systems in plants, are used to remove reactive oxygen species free radicals, relieve membrane lipid peroxidation of cells, and reduce the damage to plants caused by the hostile environment [50].In the experiment, the difference of enzyme activity between different concentrations of organic-inorganic complex fertilizers was highly significant. From the point of view of the activity size, if the concentration was too low, the promotion of enzyme activity in 'Qi-Nan' seedlings was not obvious, and if the concentration was too high, the enzyme activity was inhibited by the fertilizers, so the reasonable amount of organic-inorganic complex fertilizers could help to increase the enzyme activity in 'Qi-Nan' seedlings and enhance the seedling's resistance.

6 Conclusions

'Qi-Nan' agarwood is the highest quality and value agarwood on the market. However, no one has studied the suitable fertilization method for potted seedlings. Our study found that organic-inorganic complex fertilizer have a greater effect on the growth and development of 'Qi-Nan' seedlings, and that moderate amounts of organic-inorganic complex fertilizers will promote the growth of 'Qi-Nan' seedlings, while excessive amounts will have an inhibitory effect on their growth. Correlation analysis showed that the main factors affecting the seedlings' heights and diameters were POD, chlorophyll, and SS content, and all three of them had higher content around November, indicating that 'Qi-Nan' potted seedlings had strong photosynthetic capacity and better growth in November. Overall, the optimal fertilizer amount of ' Qi-Nan ' agarwood potted seedlings is about 7-11g/ plant. However, the results obtained from the pot trials were limited and not sufficiently representative of long-term fertilization effects. Therefore, it is recommended to follow up with additional field trials of 'Qi-Nan' fertilization and continue the research so that the results of the trials will be of practical significance.

Author Contributions: J.H., Z.Y. carried out experimental work, data analysis, interpretation and manuscript writing, these authors have contributed equally to this work.; S.Z., X.L., F.T. assisted

for pot trials, P.W., F.Y. supervised the work and contributed to the manuscript writing. All authors have read and agreed to the published version of the manuscript.

Funding: The research was funded by the Department of Forestry of Guangxi Zhuang Autonmous Region, Guangxi Forestry Science and Technology Project (Guilin Kezi [2021]9).

Data Availability Statement: All data are contained within the article.

Acknowledgments: The authors would like to thank Guangxi Miaoran Agricultural Chemical Technology Co., LTD for their supply of organic-inorganic complex fertilizers, thank the agarwood cultivation base in Nalou Town for providing the environment for pot experiment, and thank relevant staff for helping to cultivate the 'Qi-Nan' agarwood seedlings and measuring the data.

Conflicts of Interest: The authors declare no conflict of interest.

References

1. Ng, L. T. A review on agar (gaharu) producing *Aquilaria* species.J. *Tropic. Forest Prod*. **1997**, 2, 272-285.
2. Barden, A., Anak, N. A., Mulliken, T., Song, M. Heart of the matter: agarwood use and trade and CITES implementation for Aquilaria malaccensis. *Traffic International, Cambridge, UK*. **2000**, 39, 937-950.
3. Persoon, G.A.; van Beek, H.H. Growing 'the wood of the gods': Agarwood production in southeast Asia. *Smallhold. Tree Grow. Rural. Dev. Environ. Services. Springer Dordr*. **2008**, 2, 245–262.
4. Snelder, D.J.; Rodel, D.L. Smallholder Tree Growing for Rural Development and Environmental Services: Lessons from Asia; *Springer Science & Business Media: Berlin/Heidelberg, Germany*, **2008**.
5. Yamagata, E.; Yoneda, K. Pharmacognostical studies on the crude drug of agarwood (VI): On "Kanankoh". *Shoyakugaku Zasshi*, **1987**, 41, 142–146.
6. Nakanishi, T.; Inada, A.; Nishi, M.; Yamagata, E.; Yoneda, K. A new and a known derivatives of 2-(2-phenylethyl) chromone from a kind of agarwood ("Kanankoh", in Japanese) originating from *Aquilaria agallocha. J. Nat. Prod*. **1986**, 49, 1106–1108.
7. Azren, P.D.; Lee, S.Y.; Emang, D.; Mohamed, R. History and perspectives of induction technology for agarwood production from cultivated Aquilaria in Asia: A review. *J. For. Res*. **2019**, 30, 1–11.
8. Naef, R. The volatile and semi-volatile constituents of agarwood, the infected heartwood of *Aquilaria species*: A review. *Flavour Fragr. J*. **2011**, 26, 73–87.
9. Liu, J.; Yang, J.; Jiang, C.; Zhou, J.; Zhao, Y.; Huang, L. Volatile organic compound and endogenous phytohormone characteristics during callus browning in *Aquilaria sinensis. Ind. Crops Prod*. **2021**, 168, 113605.
10. Convention on Internal Trade in Endangered Species of Wild Fauna and Flora. Available online: https://checklist.cites.org (accessed on 8 April 2021).
11. Zhang, P., Li, X., Cui, Z., Xu, D. Morphological, physiological, biochemical and molecular analyses reveal wound-ing-induced agarwood formation mechanism in two types of *Aquilaria sinensis* (Lour.) Spreng. *Industrial Crops and Products*, **2022**, 178, 114603.
12. Liu, X.; Wang, Y.; Wang, H.; Wang, L.; Mei, W.; Dai, H.; Wang, J. GC-MS Analysis of Chemical Constituents of Volatile Oil from Four Kinds of Agarwood Tree Leaves. *Chinese Journal of Tropical Crops*, **2022**, 43(1), 196.
13. Wang, Y.G.; Wang, J.; Yang, J.L.; Cai, C.H.; Gai, C.J.; Mei, W.L.; Dai, H.F. Bud Grafting for Propagation of *Aquilaria sinensis* 'Reke2'. *Chin. J. Trop. Agri*. **2020**, 40, 79–88.
14. Yu, M.; Liu, Y.; Feng, J.; Chen, D.; Yang, Y.; Liu, P.; Wei, J. Remarkable Phytochemical Characteristics of Chi-Nan Agarwood Induced from New-Found Chi-Nan Germplasm of *Aquilaria sinensis* Compared with Ordinary Agarwood. *Int. J. Anal. Chem*. **2021**, 33, 533–542.
15 Liu, J.N.; Whang, L.M.; Zeng, L.M.; Liu, D.Q. Agarwood planting promotes rural revitalization in hainan Province. *J. Agric. Catastrophol*. **2021**, 11, 143–144.
16. Liu, X.J.; Xu, D.P. Characteristics of resource distribution, industry status and development proposal of precious tree species in Guangdong. *Guangdong Agric. Sci*. **2021**, 48, 57–65.
17. Fan, H.Y.; Huang, Y.; Deng, Q.H.; Song, S.Q. Agarwood family. *Life World*, **2021**, 380, 12–17.
18. Zhu, Y.; Dumroese, R.K.; Pinto, J.R.; Li, G.L.; Liu, Y. Fall fertilization enhanced nitrogen storage and translocation in Larix olgensis seedlings. *New For*. **2013**, 44, 849–861.
19. Kaiyou G. Industrialization of Organic Fertilizer and the Sustainable Agriculture Development in China[J]. *Chinese Agricultural Science Bulletin*, **2004**.
20. Ping L U, Nai-He M, Bo L. Requirement in Plant Nutrition and Fertilizer use of Organic Agriculture[J]. *Review of China Agricultural Science and Technology*, **2004**.
21. Watimena S F. Effect of Organic and Inorganic Fertilizer Application on Soybeans Growth and Yield (Glycine max L. Merril) [J]. J*ournal of Science and Science Education*, **2023**.DOI:10.29303/jossed. v4i1.3340
22. Singh A, Prasad V M, Bahadur V, & Topno, S. E. Effect of organic and inorganic fertilizer on growth, yield and quality traits of cucumber (Cucumis sativus) under Prayagraj Agro-climatic condition[J] *AkiNik Publications*, **2021**(7)
23. Mondal, A. B., Azad, A. K., Ahmed, M. B., Mannan, A., Hossain, M., & Eaton, T. E. Influence of organic and inorganic fertilizers on floating bed cultivation of okra and cucumber during summer season in southern part of bangladesh. *American Journal of Botany*, **2022**(005), 013.

24. Amfo, B., & Ali, E. B. Beyond adoption: the interaction between organic and inorganic fertilizer application, and vegetable productivity in ghana. *Renewable Agriculture and Food Systems*, **2021**, 1-17. 15
Liu, J.N.; Whang, L.M.; Zeng, L.M.; Liu, D.Q. Agarwood planting promotes rural revitalization in hainan Province. *J. Agric. Catastrophol*. **2021**, 11, 143–144.
25. Sun, G. Experimental study on the correlation between different fertilization levels and the growth of young Cinnamomum camphora forest[J]. *Journal of Green Science and Technology*, **2019**(13):3.
26. Ji, Y.Z. Effects of Fertilization on Soil Fertility and Physiological Activity of Larix gmelinii Plantation[D], Northeast Forestry University, China, **2002**.
27. Tan, C.Q.; Zhou, J.Q.; Wang, L H.; Yin, T.; Tang. C.H.; Liang, X. Comparative experiment of different eucalyptus compound fertilizer base fertilizers[J] *Forestry science and technology development*. **2014**.
28. Pang, S.J.; Zhang, P.; Yang, B.G.; Liu, S.L.; Feng, C.L. Effects of suitable ratio of biological fertilizer and chemical fertilizer on growth of Aquilaria sinensis and soil nutrients. *Soil Fertil. Sci.* **2020**, 3, 6.
29. Arunakumara, K.; Walpola, B.; Karunatunga, C. Assessment of the early growth of agarwood (*Aquilaria crassna*) seedlings under different sources of nutrients. J. *For. Sci*. **2022**, 68, 1–7.
30. Wang, R.; He, Q.; Ding, X.G.; Li, J.Y.; Zhang, F.Q.; Zhu, B.Z.; Su, Y. Effects of exponential fertilization on seedling growth and photosynthesis of Aquilarias spp. J. *Beijing For. Univ*. **2011**, 33, 7.
31. Wenjun D. Comparative study on the physical and chemical properties of eucalyptus plantation soil in Guangxi Karst and red soil area[J]. *Ecology and Environmental Sciences*, **2013**.
32. Yang, M.W. Study on Rapid Determination of Chlorophyll Content of Leaves. *Chin. J. Spectrosc. Lab*. **2002**, 19, 478–481.
33. Cai, Y.P. *Plant Physiology Experiment Instruction*; China Agricultural University Press: Beijing, China, 2014; pp. 166–170.
34. Hao, Z.B.; Cang, J.; Xu, Z. *Plant Physiology Experiment*; Harbin Institute of Technology Press: Harbin, China, 2004.
35. Li, Z.F.; Wu, X.D. Experimental Design Scheme for the Effect of Drought Stress on Content of Malondialdehyde of Indoor Ornamental Plants. *Tianjin Agric. Sci*. 2016, 22, 3.
36. Zhang, Z.A.; Chen, Z.Y. *Experimental Techniques in Plant Physiology*; Liaoning Science and Technology Press: Shenyang, China, 2008.
37. Masayuki Y, Toshihiko H. A Canopy Photosynthesis Model for the Dynamics of Size Structure and Self-thinning in Plant Populations[J]. *Annals of Botany*, **1992**(4):305-316.
38. Mazeh, M., Almadi, L., Paoletti, A., Cinosi, N., Daher, E., & Tucci, M. Use of an Organic Fertilizer Also Having a Biostimulant Action to Promote the Growth of Young Olive Trees[J]. **2021**.
39. Dai, L. L.; Shi, F. Q.; Liu, Y. Y.; Yuan, L. Z.; &Xu, Y. M. Topdressing experiment of forest plantation of *Aquilaria yunnanensis*[J], *Jounal of Shandong Forestry Science and Technology*. **2017**. 47(3), 3.
40. Feng, H. Y.; Li, X.; Xia, S. Z. Effects of different fertilization on the growth of Corylus heterophylla seedlings[J]. *Forest By-Product and Speciality in China*. **2023**(1):15-17.
41. Xingbiao, W., Tengfei, D., Shiqi, L., & Liming, J. Effects of formula fertilization on leaf nutrient dynamics and yield of sapindus mukorossi gaertn. *Journal of Nanjing Forestry University (Natural Sciences Edition)*.**2018**.
42. Xiao D, Liu X, Yang, R., Tan, Y., & Wang, K. Nitrogen input through chemical and biological pathway affects arbuscular mycorrhizal fungal communities in forage grass cultivation in southwest China[J]. *Applied Soil Ecology*, **2019**, 150:103454
43. Hao-Tian, W. U., Nie, J., Yang, W. J., Zhang, Z. Y., Kang-Hong, W. U., & Xiao-Yu, L. I. Effects of machine sowing depth and amounts of seeds and fertilizer on lodging and yield of Tartary buckwheat[J]. *Acta Prataculturae Sinica*, **2020**, 29(12):61-72.
44. Otieno P C, Nyalala S, Wolukau J. Postharvest Quality and Safety of Potted Greenhouse Tomato Grown on Forest Soil-Biosolids substrate,Blended with NPK Fertilizer[J]. *NASS Journal of Agricultural Sciences*, **2020**, 2(2):13.
45. Shi Y W, Wang Y L, Wen-Bing L I, et al. Effects of Water Stress on Soluble Protein,Soluble Sugar and Proline Content in Tamarix hispida[J]. *Journal of Xinjiang Agricultural University*, **2007**.
46. Watanabe S, Kojima K, Ide Y, et al. Effects of saline and osmotic stress on proline and sugar accumulation in Populus euphratica in vitro[J]. *Plant Cell Tissue & Organ Culture*, **2000**,63(3):199-206.
47. Li, Y.; Chen, Z. L. Wang, J. Effects of salt stress on growth and concentration of osmoregulatory substances of Suaeda heteropptera Kitagawa. *Chinese Journal of Ecology*, **2011**, 30(01):72-76.
48. Zhangliang, Jiahao, Ruiping. Physiological performance of three calcifying green macroalgae Halimeda species in response to altered seawater temperatures[J]. *ActaOceanologicaSinica*, **2020**(2):89-100.
49. Kabuto H, Hasuike S, Minagawa N. Effects of bisphenol A on the metabolisms of active oxygen species in mouse tissues[J]. *Environmental Research*, **2003**,93(1):31-35.
50. Wan, S.; Ling, Q.; Yu, T. Effect of Water Stress on POD SOD Activity and iso-POD SOD in Chinese Chestnut seedlings Leaves[J]. Beijing Agric. Coll. 1997, 12, 20–25.
