# Supplementary material for: Effects of organic-inorganic complex fertilizer on the growth and physiological characteristics of ‘Qi-Nan’ agarwood from Aquilaria sinensis (Lour.)
Source: PLoS One. 2025 Apr 4;20(4):e0320766. doi: 10.1371/journal.pone.0320766 (PMC11970636; doi:10.1371/journal.pone.0320766)
Supplement: Attachment — Submitted filename: Response to Reviews.docx [file pone.0320766.s004.docx]

Dear Editor,

Thank you and the reviewers for the comments and suggestions on how to revise the manuscript (Manuscript Number: PONE-D-24-45249) entitled “*Effects of Organic-inorganic Complex Fertilizer on the Growth and Physiological Characteristics of 'Qi-Nan' Agarwood from Aquilaria sinensis (Lour.)*”. We are truly grateful to the your comments and suggestions on improving the manuscript, which indeed help us greatly to improve the quality of our paper. Based on these comments and suggestions, we have carefully revised the content and language of the manuscript. The responses to you are presented one by one below.

If any questions remain, please let us know. We are looking forward to hearing good news from you. Thanks for your attention and patience!

Best regards,

Authors

Below are our point-by-point responses to your comments/questions.

***Response to editor:***

1. Please ensure that your manuscript meets PLOS ONE's style requirements, including those for file naming.

***Re:*** Thank you for your suggestions on the manuscript. We have carefully modified the format of the full text according to your requirements and reference templates.

2. We note that the grant information you provided in the ‘Funding Information’ and ‘Financial Disclosure’ sections do not match.

When you resubmit, please ensure that you provide the correct grant numbers for the awards you received for your study in the ‘Funding Information’ section.

***Re:*** We are very sorry for the mistake caused by our negligence. when I submitted my manuscript, I selected No Funding in the system. For this, I apologize for any inconvenience caused to you. The correction is as follows:

There are two funding projects for this study, namely:

The name of the first funding project is: Department of Forestry of Guangxi Zhuang Autonmous Region, Guangxi Forestry Science and Technology Project (Guilin Kezi [2021]9)

The grant number is: Guilin Kezi [2021]9

Funder: Department of Forestry of Guangxi Zhuang Autonmous Region, Guangxi Forestry Science and Technology Project.

and the second grant project is called: Department of Forestry of Guangxi Zhuang Autonmous Region, the Guangxi Forestry Science and Technology Project (Guilin Scientific Research [2022ZC] No. 91),

The grant number is: Guilin Scientific Research [2022ZC] No. 91.

Funder: Department of Forestry of Guangxi Zhuang Autonmous Region, Guangxi Forestry Science and Technology Project.

The funding information was also updated in the "Funding" section of the article.

3. Thank you for stating the following financial disclosure:

“The author(s) received no specific funding for this work.”

At this time, please address the following queries:

a) Please clarify the sources of funding (financial or material support) for your study. List the grants or organizations that supported your study, including funding received from your institution.

b) State what role the funders took in the study. If the funders had no role in your study, please state: “The funders had no role in study design, data collection and analysis, decision to publish, or preparation of the manuscript.”

c) If any authors received a salary from any of your funders, please state which authors and which funders.

d) If you did not receive any funding for this study, please state: “The authors received no specific funding for this work.”

Please include your amended statements within your cover letter; we will change the online submission form on your behalf.

***Re:*** We are very sorry for the mistake caused by our negligence. As with the answer to the second question, the specific funding for this study is: the Guangxi Forestry Science and Technology Project (Guilin Kezi [2021]9) and the Guangxi Forestry Science and Technology Project (Guilin Scientific Research [2022ZC] No. 91).

4. We note that your Data Availability Statement is currently as follows: All relevant data are within the manuscript and its Supporting Information files.

Please confirm at this time whether or not your submission contains all raw data required to replicate the results of your study. Authors must share the “minimal data set” for their submission. PLOS defines the minimal data set to consist of the data required to replicate all study findings reported in the article, as well as related metadata and methods.

***Re:*** We apologize for the error caused by our oversight and all relevant data is in the manuscript and its Supporting Information file, which has been uploaded as a separate file labeled "Supporting Information".

***Response to reviewer:***

**Reviewer 1**

1.The manuscript gives a good impact on the effect of various organic and inorganic fertilizers on the valuable agarwood seedlings. Valuable data was collected and presented in tables and figures, however, it lacks some statistical significance tests. It is recommended to use and apply some statistical analysis to check the the difference between treatments. e.g.Table 1 and Table 2 should be supported horizontally each row with one way ANOVA to check the difference between the treatment groups, and vertically with either repeated measure ANOVA and/or Pearson's correlation. the change in seedling height with time should be presented at least by (r) Pearson's correlation coefficient and two-tailed significance test. Moreover, the overall table could be tested with two way repeated measure ANOVA (Factor1= treatment, Factor2= Time)

***Re:*** Thank you for your patience in reviewing the manuscript and for your valuable comments. According to what you said, we have applied some statistical analysis to check the differences between the treatments, and at the same time added multiple comparisons between the treatments and the main effect of the months on the growth amount of seedling height and ground diameter, so as to make the statistical analysis more suitable and strict. The modified table and new contents are as follows. Thank you again for your valuable advice.

**3.1.1. Effects of organic-inorganic complex fertilizer on seedling height of seedlings**

**Tab 1. Dynamics of seedling height growth of ‘Qi-Nan’ agarwood seedlings under different fertilization rates (Units: cm)**

| **Month** | **Treatment** | | | | | | | **ANOVA** |
| --- | --- | --- | --- | --- | --- | --- | --- | --- |
|  | **CK** | **1** | **2** | **3** | **4** | **5** | **6** |  |
| Nov. | 17.9 | 17.2 | 17.1 | 17.5 | 17.8 | 18 | 17.7 | >0.05 |
| Dec. | 18.2 | 17.6 | 17.7 | 18.5 | 18.9 | 18.1 | 17.9 | <0.05 |
| Jan. | 18.9 | 17.9 | 18.1 | 19.6 | 20.5 | 18.4 | 18.4 | <0.05 |
| Feb. | 20 | 18.9 | 19.4 | 20.7 | 22.6 | 19.4 | 19.5 | <0.001 |
| Mar. | 21.3 | 20.7 | 23.1 | 23.6 | 24.6 | 21.7 | 21.1 | <0.001 |
| Apr. | 22.9 | 23.8 | 27.1 | 26.4 | 27.9 | 24 | 22.6 | <0.001 |
| May. | 25.2 | 28.9 | 32 | 29.6 | 30.9 | 27.3 | 24.9 | <0.001 |
| Jun. | 30.7 | 37.8 | 40.6 | 36.6 | 35.4 | 35.4 | 33.8 | <0.001 |
| Jul. | 39.5 | 46.3 | 53.7 | 49.4 | 43.7 | 46.9 | 43.2 | <0.001 |
| Aug. | 46.7 | 56 | 65.7 | 59.7 | 51.4 | 56 | 52.2 | <0.001 |
| Sep. | 53.5 | 65.4 | 76.5 | 69.3 | 60.6 | 64.7 | 61.1 | <0.001 |
| Oct. | 55.6 | 68.4 | 80.6 | 72.9 | 64 | 68.4 | 64.1 | <0.001 |
| Nov. | 56.6 | 69.6 | 81.8 | 74.1 | 65.1 | 69.5 | 65.2 | <0.001 |
| Total increment | 38.7±3.2De | 52.4±4.9Cbc | 64.7±5.6Aa | 56.6±3.2Bb | 47.3±4.2Ccd | 51.5±5.0Cbd | 47.5±3.8Ccd | <0.001 |
| Relative increment | 100.00% | 135.40% | 167.30% | 146.40% | 122.50% | 133.30% | 122.70% | <0.001 |
| P | 0.00 | 0.00 | 0.00 | 0.00 | 0.00 | 0.00 | 0.00 |  |

Note: The data in the table are mean ± standard deviation; Different capital letters indicate highly significant differences between the same index, the same period, and different treatments at the 0.01 level (P < 0.01), and different lowercase letters indicate significant differences between the same index, the same period, and different treatments at the 0.05 level (P < 0.05).

Multiple comparisons of treatment can be seen from Table 2. As the concentration of organic-inorganic complex fertilizer increases, it is helpful for the height growth of ‘Qi-Nan’ agarwood. The seedling height growth of treatment 2 is the largest, followed by treatment 3, and then the seedling height growth gradually decreases with the increase of fertilization concentration. The concentration of organic-inorganic complex fertilizer that can promote the seedling height ranges from 7-11g/ plant.

The multiple comparisons of the main effect of months in Table 3 show that the monthly growth of seedling height first increases and then decreases with the fertilization time. Among them, the differences between each month are extremely significant, and the growth of seedling height is the largest in August.

**Tab. 2 Multiple-factor comparison of treatments main effects on plant height growth of month**

| **Treatment** | **CK** | **1** | **2** | **3** | **4** | **5** | **6** |
| --- | --- | --- | --- | --- | --- | --- | --- |
| PLH | 3.22Dd | 4.36BCbc | 5.39Aa | 4.72Bb | 3.94Cc | 4.29BCbc | 3.96Cc |

**Tab. 3 Multiple comparison of monthly main effect on plant height growth of ‘Qi-Nan ’**

| **Treatment** | **Feb.** | **May.** | **Aug.** | **Nov.** |
| --- | --- | --- | --- | --- |
| PLH | 0.84Dd | 2.82Cc | 8.90Aa | 4.51Bb |

In conclusion, different concentrations of organic and inorganic compound fertilizer have significant differences on seedling height, and the optimal concentration of organic and inorganic compound fertilizer topdressing for seedling height growth of ‘Qi-Nan’ agarwood is about 7-11g/ plant.

**3.1.2. Effects of organic-inorganic complex fertilizer on ground diameter of seedlings**

**Table 2. Dynamics of ground diameter growth of ‘Qi-Nan’ agarwood seedlings under different fertilization rates (Units: mm)**

| **Month** | **Treatment** | | | | | | | **ANOVA** |
| --- | --- | --- | --- | --- | --- | --- | --- | --- |
|  | **CK** | **1** | **2** | **3** | **4** | **5** | **6** |  |
| Nov. | 5.36 | 4.76 | 4.89 | 5.26 | 4.53 | 5.01 | 4.86 | <0.05 |
| Dec. | 5.42 | 4.92 | 5.2 | 5.47 | 4.87 | 5.13 | 4.93 | >0.05 |
| Jan. | 5.6 | 5.09 | 5.55 | 5.9 | 5.17 | 5.29 | 5.01 | <0.05 |
| Feb. | 5.94 | 5.44 | 6.15 | 6.74 | 5.75 | 5.59 | 5.55 | <0.001 |
| Mar. | 6.25 | 5.89 | 6.76 | 7.31 | 6.35 | 5.93 | 5.85 | <0.001 |
| Apr. | 6.4 | 6.02 | 6.99 | 7.48 | 6.55 | 6.11 | 5.97 | <0.001 |
| May. | 6.66 | 6.35 | 7.74 | 7.94 | 6.95 | 6.47 | 6.38 | <0.001 |
| Jun. | 8.2 | 8.28 | 9.79 | 9.74 | 8.48 | 8.03 | 6.8 | <0.001 |
| Jul. | 9.36 | 10.6 | 13.03 | 12.13 | 10.73 | 10.76 | 8.28 | <0.001 |
| Aug. | 10.02 | 11.56 | 14.71 | 13.26 | 11.74 | 11.52 | 9.16 | <0.001 |
| Sep. | 10.71 | 12.55 | 16.4 | 14.59 | 12.95 | 12.48 | 9.95 | <0.001 |
| Oct. | 11.45 | 13.32 | 17.98 | 15.61 | 13.82 | 13.37 | 10.73 | <0.001 |
| Nov. | 12.24 | 14.2 | 19.63 | 17.02 | 14.81 | 14.1 | 11.41 | <0.001 |
| Total increment | 6.88±0.89d | 9.44±1.01c | 14.74±1.50a | 11.76±1.24b | 10.28±1.10bc | 9.09±0.76c | 6.55±0.75d | <0.001 |
| Relative increment | 100.00% | 137.21% | 214.24% | 170.93% | 149.42% | 132.12% | 95.20% | <0.001 |
| P | 0.00 | 0.00 | 0.00 | 0.00 | 0.00 | 0.00 | 0.00 |  |

Note: The data in the table are mean ± standard deviation; Different capital letters indicate highly significant differences between the same index, the same period, and different treatments at the 0.01 level (P < 0.01), and different lowercase letters indicate significant differences between the same index, the same period, and different treatments at the 0.05 level (P < 0.05).

From Table 4, multiple comparisons of the main effects indicated that with the increase in the topdressing amount of organic-inorganic compound fertilizers, the ground diameter growth of ‘Qi-Nan’ agarwood seedlings initially ascended and then declined. A high concentration of organic-inorganic compound fertilizers inhibited the growth of seedlings and even severely hampered the growth of seedling diameter. Among them, the seedling diameter growth was the highest under treatment 2, suggesting that a topdressing amount of 7g/plant was more conducive to the seedling diameter growth of ‘Qi-Nan’ agarwood.

From Table 5, the monthly growth of the ground diameter displayed a trend of initially rising and subsequently falling with the fertilization time. There was no significant difference between February and May, yet a highly significant difference occurred between August and November. The disparity between August and November was also highly significant. These results imply that the growth of the ground diameter accelerates in August, then slows down and essentially stops in November.

**Tab.4 Multiple-factor comparison of treatments main effects on diameter growth of month**

| **Treatment** | **CK** | **1** | **2** | **3** | **4** | **5** | **6** |
| --- | --- | --- | --- | --- | --- | --- | --- |
| Grd | 0.58Ccd | 0.78BCbc | 1.22Aa | 0.98ABb | 0.8BC6b | 0.76BCbcd | 0.55Cd |

**Tab.5 Multiple comparison of monthly main effect on diameter growth of ‘Qi-Nan ’**

| **Treatment** | **Feb.** | **May.** | **Aug.** | **Nov.** |
| --- | --- | --- | --- | --- |
| Grd | 0.31Cc | 0.36Cc | 1.59Aa | 1.02Bb |

In conclusion, the effects of different concentrations of organic and inorganic compound fertilizer on the soil diameter are significantly distinct, and the optimal concentration of organic and inorganic compound fertilizer for the soil diameter growth of ‘Qi-Nan’ agarwood seedlings is approximately 7g/plant.

2. In addition, some minor corrections in the manuscript, including punctuations and other typing errors, should be revised.

***RE:*** Thank you for raising our spelling and punctuation questions. We have reviewed the full text thoroughly and made changes.

3. Figure 1. caption line 181, the word Treat. would be expressed as "treatment." would be better.The figure legend isnot clear; if possible, clarify.

***RE:*** Thank you very much for examining the manuscript and asking this question. We have changed the "Treat" in Figure 1 and Figure 2 to "treatment." At the same time, we have modified the expression of the legend to make it clearer. Thank you again for your valuable advice.

4. Line 106 in the materials and Methods part, "The average seedling height was 17.60±0.33..." please clarify which was used with the average (±SD) or average (±SE)

***RE:*** Thank you for your careful examination of the manuscript.We have corrected the sentence to:The average seedling height was 17.60 ± 0.33 cm (average ± standard deviation) and the average ground diameter was 4.95 ± 0.29 mm (average ± standard deviation).

**Reviewer 2**

YES, manuscript technically sound, and do the data support the conclusions. Additon the authors provide the findings in their manuscript. Author applied statistical analysis which parallel finding conclusion. More language uses is understandable to reader.

***RE:*** Thank you so much for sparing your precious time from your busy schedule to review and comment on our research. Your professional and meticulous review has made us recognize the value of the article and has given us greater confidence in our research direction. Your recognition is an immense encouragement to us, and we will hold onto this encouragement to keep moving forward on the academic path and continue conducting relevant research. Sincere thanks to you once again!

**Reviewer 3**

There are some concerns about present manuscript as follow: the aquired data is belonged to one year study and so is not statistically more reliable. Moreover, There is noy any other external factor which studied beside fertilizer tretment.

***RE:*** Thank you for your careful examination of the manuscript.

The design of our trial cycle was founded on some comparable literature that has been reported, and the experimental outcomes demonstrated similar results or trends to them. For instance, some researchers selected leaves on the 67th day after fertilization for the determination of physiological indicators (Kuanysh K， Ji D D， Kuanysh K， Hui W B， Li M L. Effects of Five Foliar Fertilizers on the Growth and Physiological Characteristics of Dahongpao Prickly Ash. Journal of Northwest Forestry University.2022;37 (4).135-142. DOI:10.3969/j.issn.1001-7461.2022.04.18), and some others measured indicators on the 80th day after fertilization(HE Jinjin, DENG Tiantian, LIU Qianyu, et al. Effects of different fertilization treatments on growth and physiological characteristics of Ardisia japonica[J]. Ecological Science, 2023, 42(4): 92–97). This indicates that our results are also compelling. However, you mentioned that due to the short test duration, the obtained results have certain limitations and are insufficient to represent the long-term fertilization effect. In the future, the test period will be prolonged, and further research will be conducted to make the test results more practical.

Regarding other factors that were not considered, we mainly utilized the pot control experiment to explore the influence of fertilizer on its growth. Other factors could almost be disregarded in this experiment, and we will also carry out the relevant research you proposed in the subsequent experiment.

Thank you again for your advice and we will be more careful with our trial design in future studies.
